# Rif1 regulates telomere length through conserved HEAT repeats

**DOI:** 10.1093/nar/gkab206

**Published:** 2021-03-27

**Authors:** Calla B Shubin, Rini Mayangsari, Ariel D Swett, Carol W Greider

**Affiliations:** Department of Molecular Biology and Genetics, Johns Hopkins University School of Medicine, Baltimore, MD 21205, USA; Biochemistry, Cellular and Molecular Biology Graduate Program, Johns Hopkins University School of Medicine, Baltimore, MD 21205, USA; Department of Molecular Biology and Genetics, Johns Hopkins University School of Medicine, Baltimore, MD 21205, USA; Department of Molecular Cell and Developmental Biology, University of California, Santa Cruz, CA 95064, USA; Department of Molecular Biology and Genetics, Johns Hopkins University School of Medicine, Baltimore, MD 21205, USA; Department of Molecular Biology and Genetics, Johns Hopkins University School of Medicine, Baltimore, MD 21205, USA; Department of Molecular Cell and Developmental Biology, University of California, Santa Cruz, CA 95064, USA

## Abstract

In budding yeast, Rif1 negatively regulates telomere length, but the mechanism of this regulation has remained elusive. Previous work identified several functional domains of Rif1, but none of these has been shown to mediate telomere length. To define Rif1 domains responsible for telomere regulation, we localized truncations of Rif1 to a single specific telomere and measured telomere length of that telomere compared to bulk telomeres. We found that a domain in the N-terminus containing HEAT repeats, Rif1_177–996_, was sufficient for length regulation when tethered to the telomere. Charged residues in this region were previously proposed to mediate DNA binding. We found that mutation of these residues disrupted telomere length regulation even when Rif1 was tethered to the telomere. Mutation of other conserved residues in this region, which were not predicted to interact with DNA, also disrupted telomere length maintenance, while mutation of conserved residues distal to this region did not. Our data suggest that conserved amino acids in the region from 436 to 577 play a functional role in telomere length regulation, which is separate from their proposed DNA binding function. We propose that the Rif1 HEAT repeats region represents a protein-protein binding interface that mediates telomere length regulation.

## INTRODUCTION

Telomeres contain repetitive DNA that protects the ends of linear chromosomes in eukaryotes and allows for telomere length maintenance. Telomeres shorten due to the end replication problem. To counteract this shortening, telomerase adds telomere repeats and establishes an equilibrium length, which varies by species. Telomere binding proteins help maintain this equilibrium by positively or negatively regulating telomere addition (1,2). When the length equilibrium is disrupted, short telomeres can signal a DNA damage response, resulting in cellular senescence or cell death (3). Maintenance of telomere length equilibrium is critical for human health; short telomeres can cause age-related degenerative diseases, including pulmonary fibrosis, immune deficiency and bone marrow failure; conversely, long telomeres lead to a predisposition to cancer (4,5). Identifying the mechanistic basis of telomere length regulation is therefore important to understand the role of telomeres in disease. Here we focus on the protein Rif1, which regulates telomere length equilibrium in yeast.

**Figure 1.**
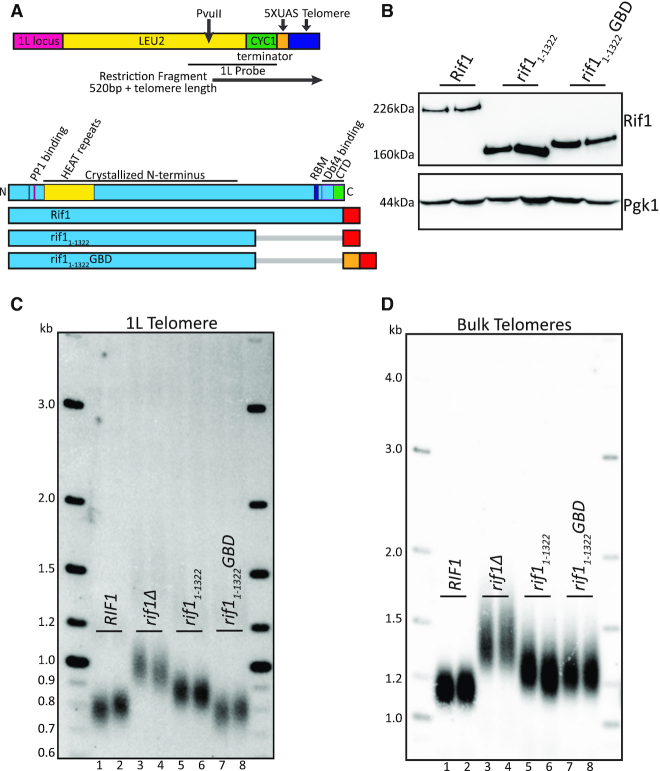
Rif1 N-terminus when localized to telomere is functionally sufficient in telomere regulation. (**A**) Diagram of 5XUAS landing pad and evaluated Rif1 constructs. The 1L telomere restriction fragment is indicated by the black arrow below the diagram beginning at the PvuII cut-site. The lower section of the diagram shows a Rif1 domain map depicted to scale (RBM: Rap1 binding motif; CTD: Carboxyl-terminal domain; Dbf4 binding overlaps CTD). Below is a schematic of the Rif1 constructs tested in this figure (Blue: Rif1; Red: 6xFLAG; Orange: GBD; Gray bar: C-terminal truncation of residues). (**B**) Western blot showing Rif1 (anti-FLAG antibody) and Pgk1 (anti-Pgk1 antibody, control) protein levels of indicated strains. (**C**) Southern blot showing 1L telomere probe for the indicated strains. (**D**) Southern blot from C, rehybridized with a Y’ probe to visualize ‘bulk’ XhoI restriction fragments. For the Southern blots in (C and D), PvuII and XhoI were both used together to digest genomic DNA. The *LEU2* gene was used as a probe to detect the PvuII restriction fragment of the unique 1L telomere (denoted in Figure 1A, primers in Supplementary Table S1) (See ‘Materials and Methods’ section).

*Saccharomyces cerevisiae* Rif1 is a 1916 amino acid protein that regulates several processes, including origin firing, DNA repair and telomere length. Rif1 was discovered in yeast through its interaction with the telomere binding protein Rap1. Deletion of *RIF1* results in long telomeres, indicating that it negatively regulates telomere length (6). Rif1 has domains that bind PP1 (protein phosphatase 1), Dbf4 (the regulatory component of DDK) and Rap1 (Figure 1A). Yeast Rif1 binds to PP1 via two canonical PP1 binding motifs, RVxF and SILK, in its N-terminus. This function is conserved from yeast to mammals, but, in mammalian Rif1, these binding sites are located in the C-terminus. The Rif1-PP1 complex blocks origin firing by de-phosphorylating Mcm4 in the pre-replication complex (7–11). We recently showed that this conserved Rif1 function does not regulate telomere length in yeast (12). The Rif1 C-terminus binds to Dbf4 (7,8), and also localizes Rif1 to the telomere through the Rap1 binding motif (RBM) (13), but deletion of these domains does not lead to telomeres that are as long as *rif1Δ* (12,13). Thus, while we know several binding partners of Rif1, the domains and mechanism by which Rif1 negatively regulates telomere length in yeast remain elusive.

Rif1 also plays a role in the DNA damage response in regulating non-homologous end joining (NHEJ). Rif1 residues implicated in DNA binding are important for carrying out Rif1 NHEJ function (14). These residues are located in the most conserved region of Rif1 homologues (15). In mammalian cells, Rif1 is recruited to double-stranded breaks (DSB) by phosphorylated 53BP1 (Rad9 in yeast). ATM (Tel1 in yeast) phosphorylation of 53BP1 is required for recruitment of Rif1, which subsequently suppresses 5′ end resection at double-strand breaks (DSB). This function counteracts BRCA1-mediated homologous recombination at DSB (16–19). The precise mechanisms by which Rif1 promotes NHEJ in yeast, and whether these functions are related to those in telomere length regulation, are not yet understood.

Since the domains of Rif1 defined to date do not explain its role in regulating telomere length, we set out to identify a telomere functional domain. We tethered several independent domains of Rif1 to a unique telomere and measured telomere length at that unique telomere and also across the bulk telomere population. We found that one conserved region of HEAT repeats in the Rif1 N-terminus, aa 177–996, is sufficient to maintain telomere length regulation when tethered to the unique telomere. Mutational analysis suggests that residues within this domain, aa 436–577, may represent a protein-binding interface that promotes telomere regulation.

## MATERIALS AND METHODS

### Reagents

Enzymes used for cloning and Southern blots are all listed in Supplementary Table S1. Antibodies used are indicated in western blotting below.

### Biological resources

Yeast strains and plasmid vectors are all listed in Supplementary Table S1 and available upon request. Bacteria used for cloning are also listed in Supplementary Table S1.

### Molecular cloning

As described in (12). Plasmid and strain design were done *in silico* using SnapGene software. Standard molecular biology techniques including PCR and Gibson assembly (New England Biolabs) were used to make all plasmids and homology repair constructs for all yeast transformations. All constructs used to generate strains, including plasmids and oligos, as well as enzymes used for digest before transformation, are listed in Supplementary Table S1. All plasmid maps are available upon request. All restriction enzymes used in these studies are from New England Biolabs. All oligonucleotides and gene blocks were ordered from Integrated DNA Technologies (idtdna.com). All *RIF1* mutations were cloned at the *RIF1* genomic locus and expressed under the *RIF1* endogenous promoter using KANMX as a selectable marker.

### Gal4 DNA binding domain (GBD) 5XUAS 1L telomere

The Gal4 DNA binding site construct was designed *in silico* using SnapGene software and constructed using standard cloning techniques. BlastN (https://blast.ncbi.nlm.nih.gov/) was used to determine a unique homology arm for one-ended recombination on chromosome arm 1L upstream of the X element and other duplicated genes (CS218, Supplementary Table S1). *LEU2* was used as a selectable marker, with a silent change T1674A to create a novel PvuII cut-site for telomere Southern analysis. The *CYC1* terminator was included to prevent *LEU2* transcription into the 5XUAS landing pad. The 5XUAS-binding site was inserted directly next to a 40 bp telomere seed sequence with an I-Sce1 site, which, once cut, telomerase can recognize and extend, leading to *de novo* telomere elongation *in vivo* (20). PCR was used to amplify the construct from pCS33 while simultaneously adding 40 bp of homology (CS155 and CS218, Supplementary Table S1) to chromosome 1L for one-ended homologous recombination, and then I-Sce1 digest was used to create the 3′ overhang before standard yeast transformation as described below. Transformed yeast were plated on minimal media plates lacking leucine, supplemented with nicotinamide (NAM) at 5mM. NAM allows for selection of inserted genes in heterochromatic regions of the genome such as the telomere by counteracting Sir2 chromatin modifications to allow expression of the selectable marker (21).

### Yeast culturing and transformation

Cells were grown logarithmically in yeast peptone dextrose (YPD) to an optical density of close to 1.0 described in (12). Cells were washed and resuspended in sterile water and 0.1 M lithium acetate (LiAc, Sigma L6883–250G) before pelleting. About 50 μl of the cell pellet was used in the transformation alongside the DNA homology repair template, 0.1 M LiAc. Herring sperm DNA was added as a carrier for most transformations. The transformation reaction was incubated at 30°C for 10 min before adding 500 μl polyethylene glycol (PEG, Sigma P4338–1KG) and another incubation at 30°C for 30 min. The heat shock step was performed at 42°C for 15 min to 1 h. Cells were washed by adding sterile water to bring the volume up to 1 ml before pelleting; the wash step was often repeated with another 1 ml sterile water before plating. If selecting for a drug resistant marker such as KANMX, cells were resuspended after the wash step in 1 ml YPD and recovered at 30°C for 4 h. Oligonucleotides or enzymes used to amplify and isolate the homology repair template are listed with the strain list in Supplementary Table S1.

### Southern blotting

Genomic DNA (gDNA) was isolated by pelleting 1.5 ml saturated overnight yeast culture grown in YPD, then beating the cells using 0.5 mm glass beads in 250 μl lysis buffer and 200 μl phenol chloroform. Cells were spun down for 10 min at 14 000 rpm, and ∼200 μl of the top clear solution, containing the gDNA, was carefully taken out. gDNA was precipitated using 500 μl of 95% ethanol and then pelleted. The supernatant was discarded, and 500 μl of 70% ethanol was added. Pellets were left to dry before resuspension in 50 μl TE with RNaseA for 1 h at 37°C or overnight at 4°C. About 10 μl of gDNA was digested using PvuII and XhoI for 1–4 h at 37°C. Of note, PvuII and XhoI were both used together to digest genomic DNA for all Southern blots to visualize the 1L telomere (PvuII) and bulk telomere (XhoI) restriction fragments. To better visualize the 1L telomere, 10 ng of 2-log ladder (NEB N3200L) were loaded instead of 100 ng, as done previously (12). Digested gDNA and ladder were loaded onto a 1% agarose gel and electrophoresed overnight at ∼47V in 1X TTE. The gel was denatured for 30 min in a rocking shaker (1.5 M NaCl, 0.5 M NaOH) and was neutralized for 15 min (1.5 M NaCl, 0.5M Tris, pH 7.0). gDNA on the gel was vacuum transferred onto a hybond nylon membrane (GE Healthcare GERPN303B) with 10X SSC (1.5 M NaCl 0.17 M NaCitrate, dihydrate) and UV cross-linked before blocking in Church buffer (0.5 M Na_2_HPO_4_, pH7.2, 7% SDS, 1 mM EDTA, 1% BSA) for ∼1 h at 65°C. ^32^P radiolabeled PCR fragments were added onto the membrane and left to incubate overnight. 1L telomere Southern blots were hybridized with a radiolabeled purified PCR product of the *LEU2* gene and *CYC1* terminator amplified from a plasmid (pCS206) using primers CS414 and CS432, and bulk telomere Southern blots were hybridized with a Y’ PCR product. Oligonucleotide sequences used to generate the PCR products are listed in Supplementary Table S1. The membrane was washed 3–4 times for 15 min each, with 1X SSC 0.1%SDS buffer before laying down a phosphor screen (GE Healthcare). 1L telomere Southern blots were exposed to phosphor screens for 4–5 days and Y’ telomere Southern blots were exposed for 1 day. Images were captured on a STORM using ImageQuant software (GE Healthcare), and the .gel files were copied into PowerPoint and saved as .tif files. Telomere length was measured from at least two clones of each genotype.

### Western blotting

Protein extraction using trichloroacetic acid (TCA, Sigma T0699) and western blotting methods are similar to those described in (12). About 500 μl of cells were collected from overnight saturated yeast cultures grown in YPD. Cells were pelleted, and 1:10 ratio of TCA to water was added. Tubes were inverted to gently mix and left to incubate at room temperature for 30 min. Cells were pelleted, resuspended in 500 μl 1 M HEPES (Teknova H1035), and then centrifuged to remove the supernatant. Pellets were resuspended in 50 μl 2× LDS sample buffer (Invitrogen NP0007), supplemented with 100 mM DTT, and vortexed with ∼50 μl of 0.5 mm glass beads. About 50 μl of LDS buffer was added. The sample was boiled at 100°C for 5 min, and then spun down for 10 min, before carefully taking the supernatant. Samples were kept on ice or -20°C freezer until ready to be used and re-boiled right before loading onto the gel. Tris-acetate gels (3–8%, Invitrogen EA0375) were run at 150 V for 1 h and 20 min to resolve all proteins. Short transfer using Trans-Blot Turbo transfer system (Bio-Rad) with the pre-set 10-min-high MW program was used for all westerns unless blotting for full-length Rif1, where a long transfer using NuPAGE XCell II Blot Module (Thermofisher/Invitrogen EI9051) was used at 30 V for 1.5 h. Both αFLAG for Rif1 blots (1:1000) (Sigma M8823) and αPgk1 (1:10 000) (Invitrogen 459250) antibodies were blocked in 5% milk TBS-T (1X TBS 0.1% Tween-20). Secondary for both was αMouse (1:10 000) (Bio-Rad 1706516), also in 5% milk TBS-T. Forte HRP substrate (Millipore WBLUF0100) was used for imaging FLAG blots, and SuperSignal West Pico PLUS Chemiluminescent Substrate (Thermo 34580) was used for imaging Pgk1 blots. Signals were captured on an LAS-4000 imager (GE healthcare). Images were visualized using ImageQuant software (GE Healthcare), and the .gel files were copied into PowerPoint and saved as .tif files. Rif1 protein levels were visualized by western blot in at least two clones of each genotype.

### Protein stability

Several constructs, all expressed at the endogenous *RIF1* promoter, had unexpected changes in Rif1 protein levels that limited data interpretation. We tested a construct containing only the C-terminal region, from amino acids 1323–1916, *rif1-NLS_1323__–__1916_GBD*. Unexpectedly, this construct was highly overexpressed even at the endogenous *RIF1* promoter and ran at a much higher than expected molecular weight. We found a partial rescue of telomere length at both the 1L telomere and at bulk telomeres in this strain. Disrupting the Rap1 binding motif with a two amino acid substitution, I1762R and I1764R (13), still showed partial rescue, indicating binding to Rap1 may not be required for this C-terminal construct. While it is difficult to make a conclusion due to the high level of expression, we cannot rule out some role of the C-terminal region in regulating telomere length. In addition, we generated eight constructs containing internal deletions in Rif1 (Δ1–152, Δ191–340, Δ342–484, Δ486–684, Δ686–893, Δ895–1142, Δ1144–1221, Δ1223–1318). We found most of these constructs had low to undetectable protein levels on western blots, precluding this approach for domain mapping.

### Nuclear localization signal (NLS) identification

Potential NLS were identified with nls-mapper.iab.keio.ac.jp, using *Saccharomyces cerevisiae* Rif1 protein sequence and using a strong cut-off parameter of 7 to identify NLS consensus. The only strong predicted NLS was located at position 56 with a high score of 13.

### Structure guided sequence alignment

Structure-guided sequence alignment was generated by first employing a multiple sequence alignment (MSA) of Rif1 orthologs from 12 yeast species using Clustal Omega (http://www.clustal.org/omega/). A FASTA file that contained a compilation of the Rif1 protein sequence, obtained from Orthogroup Repository Documentation and UniProt database, was used in generating the multiple sequence alignment. The output Clustal Omega file was then uploaded to the ConSurf Server (https://consurf.tau.ac.il) with the crystal structure of the Rif1-NTD dimer in complex with DNA double helicase (PDB: 5NW5) as a reference and *S. cerevisiae* as the query sequence. The ConSurf Server generated a PDB file in which the residues were color coded based on their conservation score ranging from 1 to 9, with the score of 1 being the most variable and 9 being the most conserved. The Rif1 structure-guided alignment PDB file was then visualized and analyzed in PyMOL (The PyMOL Molecular Graphics System, Version 2.2.0, Schrödinger, LLC) (Supplementary Figure S2D). We also visualized the alignment using SnapGene software (Supplementary File S1) and graphed the conservation scores using GraphPad Prism 5.

## RESULTS

### The C-terminus of Rif1 is not required for telomere length regulation

To examine the regions of Rif1 that mediate telomere length regulation, we generated a yeast strain in which we could localize domains of Rif1 directly upstream of a unique telomere. We introduced five copies of the GAL4 upstream activating sequence, 5XUAS, immediately adjacent to telomere repeats on the left arm of chromosome 1 (1L) by eliminating the 1L subtelomeric X element (Figure 1A; ‘Materials and Methods’ section). We then fused the GAL4 DNA binding domain, GBD, to domains of Rif1 to localize them to this unique telomere (Figure 1A,B; ‘Materials and Methods’ section). We analyzed the 5XUAS 1L telomere by Southern blot with a unique probe to this region (see ‘Materials and Methods’ section). The 1L telomere showed a length distribution with a midpoint of 800 bp, which includes the telomere and ∼500 bp of subtelomere, indicating its length distribution is regulated similarly to wild-type yeast telomeres (Figure 1C, lanes 1–2). Deletion of *RIF1* resulted in elongation of the 1L telomere and bulk telomeres, as expected (Figure 1C, lanes 3–4; Figure 1D, lanes 3–4).

To begin dissecting Rif1 domains, we initially deleted the Rap1 binding site using a C-terminal truncation, *rif1_1__–__1322_*, which removes both Rap1 binding and Dbf4 binding (Figure 1A,B). We made this truncation at the *RIF1* genomic locus, and we tested *rif1_1__–__1322_* without the *GBD* fusion, so it would not be tethered to the 1L telomere. *rif1_1__–__1322_* showed long telomeres at 1L, indicating loss of telomere length regulation (Figure 1C). When the same Southern was re-hybridized with the subtelomeric Y’ probe to visualize most other telomeres (‘bulk telomeres’, see ‘Materials and Methods’ section), *rif1_1__–__1322_* bulk telomeres were also long. Notably, both the 1L and bulk telomeres were not as long as *rif1Δ* (Figure 1C, compare lanes 5–6 to 3–4; Figure 1D, compare lanes 5–6 to 3–4). This partial effect of *rif1_1__–__1322_* may be due to a lower concentration of Rif1 near the telomere when it is not bound to Rap1.

To control recruitment of Rif1 to the 1L telomere, we fused the GBD to *rif1_1__–__1322_*, to create *rif1_1__–__1322_GBD* at the *RIF1* genomic locus (Figure 1A,B). Strikingly, *rif1_1__–__1322_GBD* restored telomere length at 1L similar to wild-type length (Figure 1C, compare lanes 7–8 to 1–2). This result shows that Rif1 residues 1–1322 are fully functional when recruited to a single telomere. This rescue effect was not seen at bulk telomeres, which were long, as expected, since *rif1_1__–__1322_GBD* is not recruited to bulk telomeres (Figure 1D, compare lanes 7–8 to 5–6 to 3–4). These data indicate that the N-terminal region of Rif1, when tethered to the telomere, is sufficient for telomere length regulation. We further used this platform to probe Rif1 functional domains by comparing the effects on the single 1L telomere, to which Rif1-GBD can bind, to the global effects on bulk telomeres.

### Identification of a required nuclear localization signal in the N-terminus of Rif1

**Figure 2.**
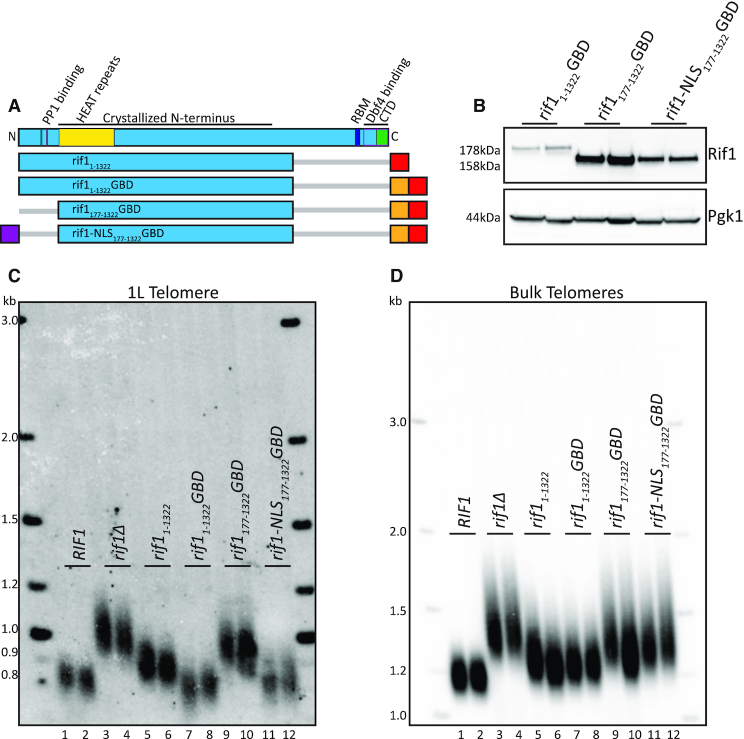
N-terminus contains critical NLS. (**A**) Rif1 domain map as in Figure 1A. Schematic of Rif1 constructs tested in this figure (Blue: Rif1; Red: 6xFLAG; Orange: GBD; Purple: c-myc NLS; Gray bar: N- or C-terminal truncation of residues). (**B**) Western blot showing Rif1 (anti-FLAG antibody) and Pgk1 (anti-Pgk1 antibody, control) protein levels of indicated strains. (**C**) Southern blot showing 1L telomere probe for the indicated strains. (**D**) Southern blot from C, rehybridized with a Y’ probe to visualize ‘bulk’ XhoI restriction fragments (see ‘Materials and Methods’ section).

Having defined the N-terminus of Rif1 as sufficient for telomere regulation when tethered by the GBD, we next sought to characterize functional domains within this region. We previously demonstrated that point mutations in the N-terminal PP1-binding site (aa 114–149) disrupted origin firing but were dispensable for telomere length regulation (12). To further probe this region, we removed 176 amino acids from the N-terminus, which we refer to as the N-terminal domain, or NTD. This NTD deletion removes the PP1-binding sites (Figure 2A). The rif1_177–1322_GBD construct (Figure 2A) was stably expressed, and, in fact, had somewhat higher steady state levels than rif1_1–1322_GBD, as indicated by western blot (Figure 2B). We found that 1L telomeres were elongated in *rif1_177__–__1322_GBD* cells, in spite of this increased protein expression (Figure 2C, lanes 9–10), indicating that the NTD has a functional role in telomere regulation. To examine this result further, we used computational analysis and found a predicted nuclear localization signal (NLS) in this region, beginning at amino acid 56 (see ‘Materials and Methods’ section). To promote nuclear localization of the fusion protein, we added c-myc NLS (22) to generate *rif1-NLS_177__–__1322_GBD* (Figure 2A,B). This construct restored the telomere length distribution of telomere 1L similar to wild-type length (Figure 2C, compare lanes 11–12 to 1–2). However, the bulk telomeres were still long, as expected, since the construct was not localized to bulk telomeres (Figure 2D, lanes 11–12 and 9–10).

We next asked whether this NLS is important in the full-length Rif1. We made an N-terminal truncation of 67 amino acids which removes the predicted NLS (Supplementary Figure S1A). We found that *rif1_68__–__1916_* has telomeres longer than wild-type, but not as long as deletion of *RIF1* (Supplementary Figure S1B, compare lanes 3–4 to 1–2 and 7–8). When we appended the N-terminal truncation with the exogenous NLS, we found *rif1-NLS_68__–__1916_* telomeres to be similar to wild type (Supplementary Figure S1B, compare lanes 5–6 to 1–2). These data suggest that the NTD delivers a critical NLS for Rif1 function, and there may also be a region in the C-terminal 1323–1916 amino acids that provides additional NLS function.

### The HEAT repeats from aa 177–996 are sufficient to maintain Rif1 telomere length function

**Figure 3.**
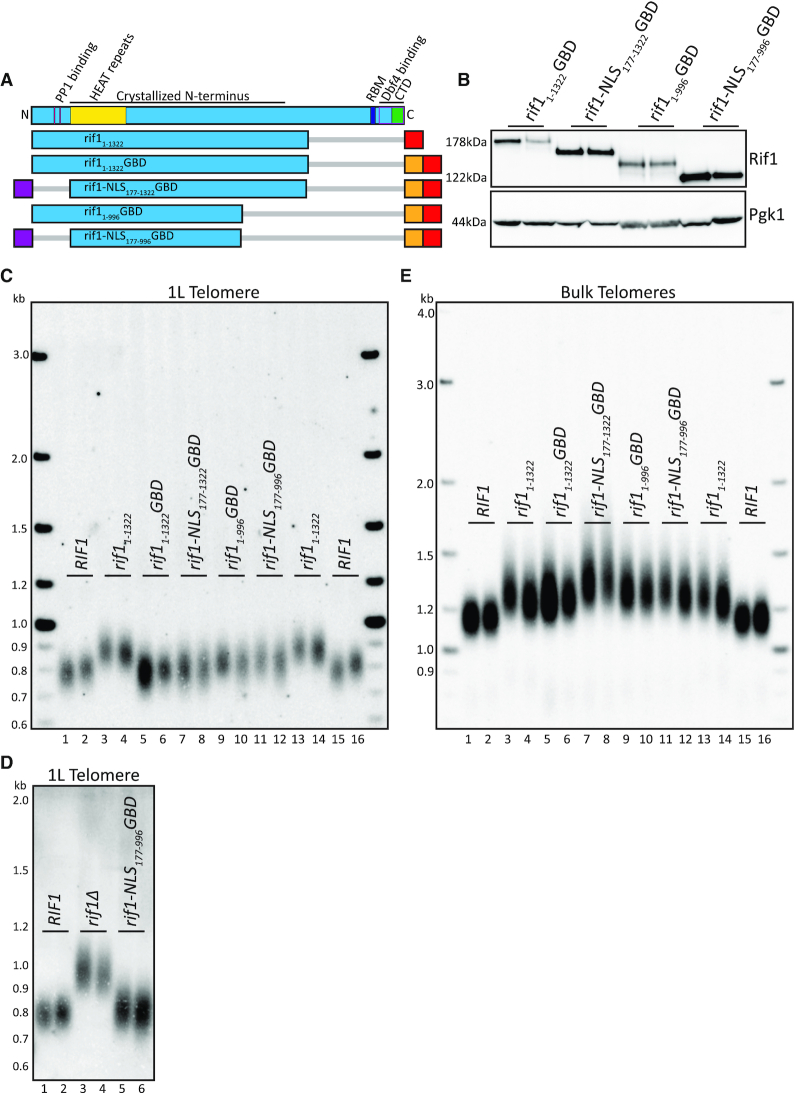
HEAT repeats of Rif1 when localized to telomere are functionally sufficient in telomere regulation. (**A**) Rif1 domain map as in Figure 1A. Schematic of Rif1 constructs tested in this figure (Blue: Rif1; Red: 6xFLAG; Orange: GBD; Purple: c-myc NLS; Gray bar: N- or C-terminal truncation of residues). (**B**) Western blot showing Rif1 (anti-FLAG antibody) and Pgk1 (anti-Pgk1 antibody, control) protein levels of indicated strains. (**C**) Southern blot showing 1L telomere probe for the indicated strains. (**D**) Southern blot showing 1L telomere probe for the indicated strains. (**E**) Southern blot from C, rehybridized with a Y’ probe to visualize ‘bulk’ XhoI restriction fragments (see ‘Materials and Methods’ section).

To further refine the functional region for length regulation, we made an additional truncation at the C-terminus. Truncation of Rif1 to residue 996 was previously shown to retain origin firing regulation activity (7). To test whether this truncation also retained telomere length regulation, we generated *rif1_1__–__996_GBD* and *rif1-NLS_177__–__996_GBD* (Figure 3A), both of which were stably expressed (Figure 3B). Both *rif1_1__–__996_GBD* and *rif1-NLS_177__–__996_GBD* were able to maintain 1L telomeres at a length similar to wild-type (Figure 3C, compare lanes 9–10 and 11–12 to 1–2 and 15–16). The smallest construct tested, *rif1-NLS_177__–__996_GBD*, was sufficient to restore length similar to wild-type at the 1L telomere length compared to cells that completely lack *RIF1* (Figure 3D). However, while *rif1-NLS_177__–__996_GBD* was sufficient to function when tethered to the telomere, bulk telomeres remained long (Figure 3E, lanes 11–12). Rif1_177–996_ contains the HOOK domain residues 185–874 defined in Mattarocci *et al.* (14). This domain is largely comprised of HEAT repeats, a helical structural motif that can promote protein–protein interactions (23) (Figure 3A). We conclude that the HEAT repeats of Rif1 residues 177–996 are sufficient for telomere length regulation when localized to the nucleus and tethered to the telomere.

### Tel1 phosphorylation is not required for HEAT repeat regulation of telomere length

**Figure 4.**
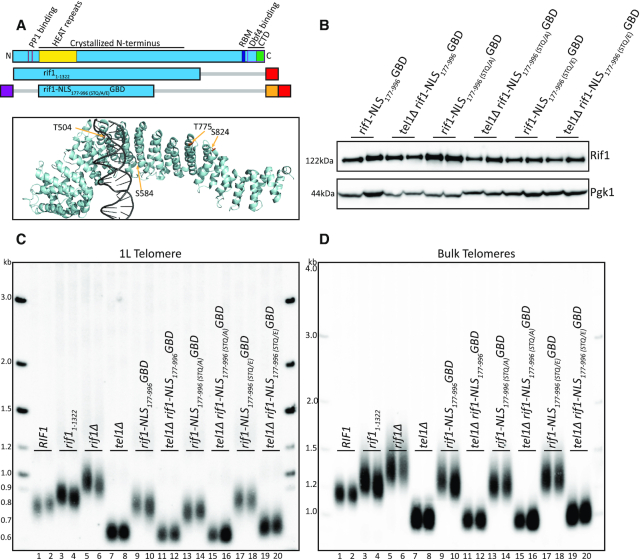
S/T-Q alanine and glutamic acid mutations have little effect on telomere length. (**A**) Rif1 domain map as in Figure 1A. Schematic of constructs tested in this figure (Blue: Rif1; Red: 6xFLAG; Orange: GBD; Purple: c-myc NLS; Gray bar: N- or C-terminal truncation of residues). PyMOL rendering of Rif1 structure with S/T-Q residues depicted in Gold (PDB: 5NW5, showing one Rif1 monomer and DNA). (**B**) Western blot showing Rif1 (anti-FLAG antibody) and Pgk1 (anti-Pgk1 antibody, control) protein levels of indicated strains. (**C**) Southern blot showing 1L telomere probe for the indicated strains. (**D**) Southern blot from C, rehybridized with a Y’ probe to visualize ‘bulk’ XhoI restriction fragments (see ‘Materials and Methods’ section).

Rif1 is a known substrate of Tel1 kinase (24–26), and genetic analysis indicates that *TEL1* and *RIF1* are in the same pathway of telomere length regulation (26,27). While mutation of a cluster of S/T-Q motifs at positions 1308–1569 to AQ in the full-length Rif1 also did not affect telomere length (26), a recent paper implicated six S/T-Q motifs in the N-terminus of Rif1 as possibly playing a role in telomere length regulation (25). We wanted to test the hypothesis that Tel1 is epistatic to Rif1 because Tel1 phosphorylates Rif1 at S/T-Q sites in the Rif1_177–996_ construct. Four of these sites are in the Rif1_177–996_ construct and are the only S/T-Q motifs in this region. To test importance of these sites in *rif1-NLS_177__–__996_GBD* function, we mutated all four S/T-Q motifs (T504, S584, T775 and S824) to either alanine (A), or to a phosphomimic glutamic acid (E) (Figure 4A,B). If the role of Tel1 in telomere length acts through phosphorylation of Rif1, we predict two outcomes for telomere length: first, the A mutant should mimic short *tel1Δ* telomeres (Figure 4C, lanes 7–8) (28); and second, the E mutant should mimic long *rif1Δ* telomeres (Figure 4C, lanes 5–6). We found that both the A and E mutants had similar telomere length to their wild-type counterpart (Figure 4C, lanes 9–10, 13–14, 17–18), suggesting that phosphorylation of these S/T-Q motifs does not play a major role in length regulation. However, we note that the A mutant had slightly shorter and the E mutant had slightly longer telomeres compared to wild-type, so we cannot exclude that Tel1 phosphorylation of these sites plays some minor role in Rif1 telomere regulation. Bulk telomeres also showed a subtle increase when comparing the A mutation to the E mutation in *rif1-NLS_177__–__996_GBD* (Figure 4D, compare lanes 13–14 to 17–18). To test whether these small effects required the presence of Tel1, we made double mutants of *tel1Δ* together with *rif1-NLS_177-__996 (STQ/A/E__)_GBD* mutants. All double mutants had short telomeres, showing that *TEL1* is epistatic to Rif1_177–996_ (Figure 4C,D, lanes 11–12, 15–16, 19–20). Together, this result and the previous mutational analysis (26) indicate that Tel1 phosphorylation of Rif1 does not play a major role in Rif1 telomere regulation.

### Positively charged residues in the HEAT repeats are required for telomere length maintenance even when tethered to the telomere

Mutations that introduce negative charges in the HEAT repeats, termed HOOK mutations, were previously shown to affect telomere length (12,14). Mattarocci *et al.* suggested that this domain mediates nonspecific binding of Rif1 to DNA via positively charged residues. Based on a crystal structure, the authors proposed that three positively charged lysine residues, K437, K563, and K570, mediate binding to the negative backbone of DNA, and they showed that a charge swap mutation to glutamic acid resulted in long telomeres (14).

**Figure 5.**
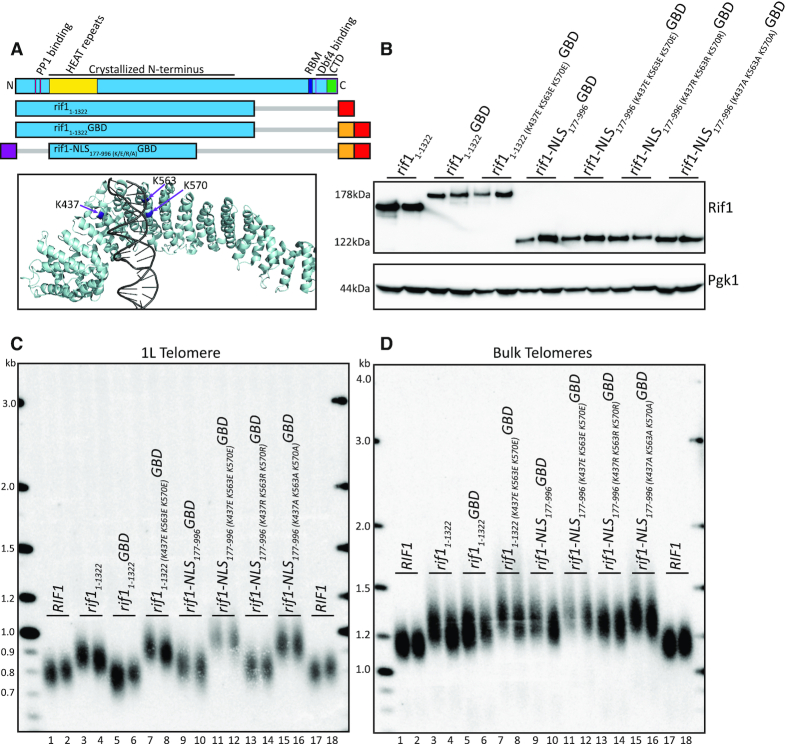
Positively charged residues in HEAT repeats are required for Rif1 function when localized to telomere. (**A**) Rif1 domain map as in Figure 1A. Schematic of constructs tested in this figure (Blue: Rif1; Red: 6xFLAG; Orange: GBD; Purple: c-myc NLS; Gray bar: N- or C-terminal truncation of residues). PyMOL rendering of Rif1 structure with lysine residues (K437, K563, K570) depicted in Purple (PDB: 5NW5, showing one Rif1 monomer and DNA). (**B**) Western blot showing Rif1 (anti-FLAG antibody) and Pgk1 (anti-Pgk1 antibody, control) protein levels of indicated strains. (**C**) Southern blot showing 1L telomere probe for the indicated strains. (**D**) Southern blot from C, rehybridized with a Y’ probe to visualize ‘bulk’ XhoI restriction fragments (see ‘Materials and Methods’ section).

To test whether the positively charged lysine residues might function to localize Rif1 to or near the telomere, or whether they might have other roles in telomere regulation, we mutated K437, K563, and K570 in our Rif1 constructs. If these lysines are mainly important for Rif1 localization to DNA, they should be dispensable at the unique 1L telomere as the GBD is sufficient to localize Rif1 to the 1L telomere. We mutated the three lysine residues, K437, K563, and K570, in both *rif1_1__–__1322_GBD* and *rif1-NLS_177__–__996_GBD*, to glutamic acid to make the same changes as in the earlier study (Figure 5A). These constructs were stably expressed (Figure 5B), but the 1L telomere length was long (Figure 5C), indicating that these mutations disrupt Rif1 telomere length function, even though the fusion protein is localized to the telomere with GBD. To further test whether the positive charge of these lysines was important, we mutated each of them to arginine, another positively charged residue, which cannot be post-translationally modified as lysine can be. We found that these arginine substitutions in *rif1-NLS_177__–__996 (K437R K563R K570R__)_GBD* allowed Rif1 regulation of 1L telomere length (Figure 5C), demonstrating that the charge of the residues, and not their possible modification, is important for Rif1 telomere function. Finally, we mutated the three lysine residues to alanine to test whether neutral charge would allow for Rif1 telomere length function. We found that 1L telomeres were long in the alanine substitution, *rif1-NLS_177__–__996 (K437A K563A K570A__)_GBD* (Figure 5C), supporting the conclusion that the positive charge of these residues is important for telomere length regulation. In all of the mutants, bulk telomeres were long as expected, since this construct does not localize to bulk telomeres (Figure 5D). Together, these results indicate that the positive charge of K437, K563, and K570 is important even when Rif1 is tethered to the 1L telomere, suggesting these residues are performing a function other than localizing Rif1 through binding DNA.

### Conserved, non-positively charged, residues in HEAT repeats regulate telomere length

To further define the role of the Rif1 HEAT repeats in telomere function, we mutated conserved residues in the *rif1-NLS_177__–__996_GBD* construct to determine if other residues also contribute to telomere function. Because Rif1 telomere function is conserved across many yeasts (29,30), conserved residues may define regions of Rif1 needed for telomere function. First, we performed a structure-guided sequence alignment of Rif1 proteins comparing 12 yeast species (‘Materials and Methods’ section; Supplementary Figure S2A,B) to identify highly conserved residues (Supplementary File 1). We found several regions of conservation throughout Rif1, similar to previous reports (13,14). These regions include the PP1 binding site and the C-terminal Rap1 and Dbf4 binding motifs (Supplementary Figure S2C,D). The NLS that we identified is also highly conserved (Supplementary Figure S2C). Strikingly, the largest region of high conservation was in the HEAT repeats, which is included within *rif1-NLS_177__–__996_GBD* (Supplementary Figure S2C).

**Figure 6.**
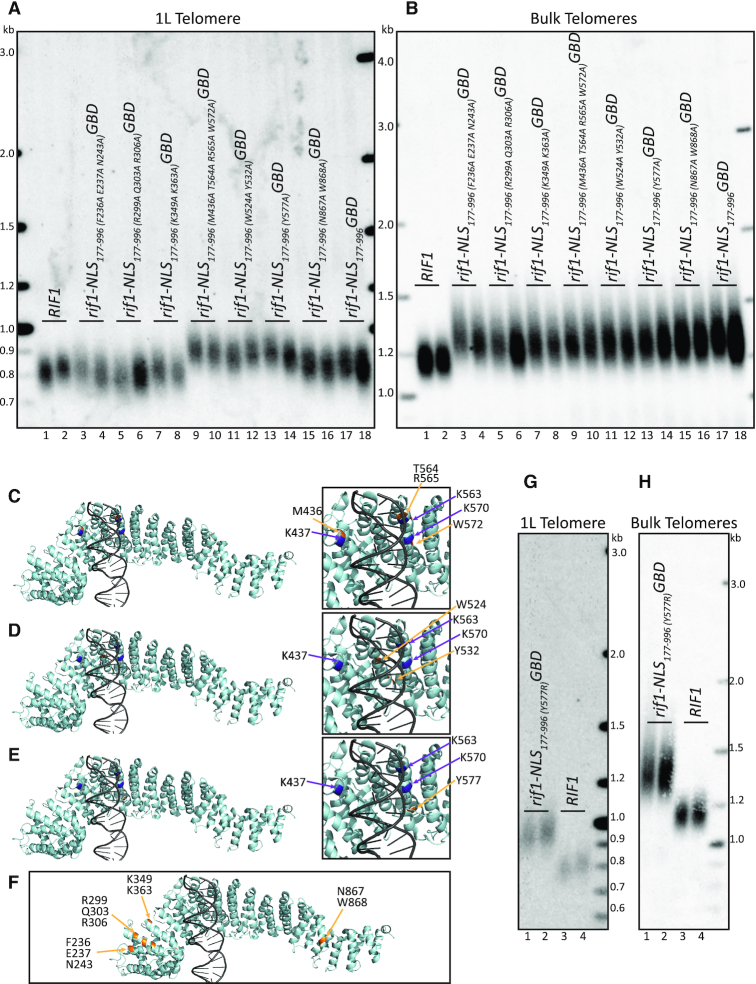
Conserved residues in HEAT repeats are critical for Rif1 function when localized to 1L telomere. (**A**) Southern blot showing 1L telomere probe for the indicated strains. (**B**) Southern blot from A, rehybridized with a Y’ probe to visualize ‘bulk’ XhoI restriction fragments (see ‘Materials and Methods’ section). (**C**) PyMOL renderings of Rif1 structure. Purple: lysines (K437, K563, K570). Gold: residues mutated in strains from lanes 9–10 in above Southern blots (M436, T564, R565, W572) (PDB: 5NW5, showing one Rif1 monomer and DNA). (**D**) PyMOL renderings of Rif1 structure. Purple: lysines (K437, K563, K570). Gold: residues mutated in strains from lanes 11–12 in above Southern blots (W524, Y532) (PDB: 5NW5, showing one Rif1 monomer and DNA). (**E**) PyMOL renderings of Rif1 structure. Purple: lysines (K437, K563, K570). Gold: residues mutated in strains from lanes 13–14 in above Southern blots (Y577) (PDB: 5NW5, showing one Rif1 monomer and DNA). (**F**) PyMOL rendering of Rif1 structure. Purple: lysines (K437, K563, K570). Gold: residues mutated in strains from lanes 3–8 and 15–16 in above Southern blots (PDB: 5NW5, showing one Rif1 monomer and DNA). (**G**) Southern blot showing 1L telomere probe of the indicated strains. (**H**) Southern blot from G, rehybridized with a Y’ probe to visualize ‘bulk’ XhoI restriction fragments (see ‘Materials and Methods’ section).

To further characterize the role of the HEAT repeats, we initially tested whether other residues in this region were functionally important. We identified conserved residues near the lysine residue cluster described above (K437, K563, K570), which were predicted not to interact with the DNA based on the crystal structure (PDB: 5NW5). We mutated these conserved residues to alanine in three groups (Figure 6A, lanes 9–14). The PyMOL rendering of the mutated conserved residues (Gold) illustrates their relative proximity to the previously described lysines, K437, K563, and K570 (purple), and distance from the DNA (gray) (Figure 6C,D,E). We first tested a mutant of four residues, M436A, T564A, R565A and W572A, each residue near one of the previously tested lysines, and found this mutant had long telomeres at the 1L telomere (Figure 6A, compare lanes 9–10 to 17–18; Figure 6C; Supplementary Figure S3A). We next mutated two residues, W524A and Y532A, which are also near the DNA but not predicted to interact with it, and also found long telomeres at the 1L telomere (Figure 6A, compare lanes 11–12 to 17–18; Figure 6D; Supplementary Figure S3A). Finally, we mutated a conserved tyrosine, Y577, which is also predicted not to interact with DNA, and again found long telomeres at the 1L telomere (Figure 6A, compare lanes 13–14 to 17–18; Figure 6E; Supplementary Figure S3A). It was striking that mutation of a single residue, Y577, had a major effect on telomere length. To further test Y577, we asked whether a positive charge in this position would allow for telomere length regulation, since we found the positive charge of the lysine cluster to be important. We mutated Y577 to arginine, and found that Y577R had long 1L telomeres, indicating that non-positively charged residues are also functionally important in this region of the protein and that additional positive charge in the region is not beneficial to function (Figure 6G; Supplementary Figure S3B). These data suggest the Rif1 conserved HEAT repeats may provide a protein binding surface, consistent with the function of other HEAT repeat domains (23).

Rif1 is also known to localize to the nuclear periphery by palmitoylation of two cysteine residues, C466 and C473, within the HEAT repeat of interest (31). We mutated these residues to alanine (Supplementary Figure S4A) and tested telomere length in full-length Rif1 (Supplementary Figure S4B). We found that mutation of C466 and C473 to alanine did not alter telomere length (Supplementary Figure S4B, compare lanes 3–4 to 5–6). We also note that deletion of *PFA4*, which is responsible for Rif1 palmitoylation, was previously shown to have wild-type telomere length (32). This result and our data together suggest that Rif1 palmitoylation is not required for the Rif1 telomere function of the HEAT repeats.

We next mutated highly conserved residues that were located distal to the previously characterized lysines (K437, K563, K570), and we found these mutations did not affect telomere length (Figure 6F; Supplementary Figure S3A). A first mutant containing three residue substitutions, F236A, E237A, and N242A, a second mutant containing three other substitutions, R299A, Q303A, and R306A, and a third mutant with two residue substitutions, K349A and K363A, all had a 1L telomere length similar to the *rif1-NLS_177__–__996_GBD* control construct (Figure 6A, compare lanes 3–4, 5–6, 7–8 to 1–2; Figure 6F), indicating they do not disrupt Rif1 function. We saw some clonal variation in both the (F236A, E237A, N242A) and (R299A, Q303A, R306A) mutants, but found the majority of these mutants showed telomere length similar to the *rif1-NLS_177__–__996_GBD* control. Our data suggest that conserved residues distal from the K437, K563, and K570 lysine residues, regardless of charge, are not important in Rif1 telomere length regulation.

The crystal structure suggests that central residues within Rif1 might mediate a dimer interface in the presence of DNA; however, Rif1 also crystalized as a monomer (PDB: 5NVR and 5NW5, respectively) (14). To test the function of this putative dimer interface on telomere regulation, we mutated the two most highly conserved residues that may mediate dimerization, N867 and W868 (Figure 6F; Supplementary Figure S3A). We found that this mutant did not disrupt Rif1 function, as 1L telomere length was similar to that of the *rif1-NLS_177__–__996_GBD* control (Figure 6A, compare lanes 15–16 to 17–18; Figure 6F). This result suggests that Rif1 dimerization may not be critical for function in telomere length regulation. The bulk telomeres of all *rif1-NLS_177__–__996_GBD* mutants tested were long, as expected (Figure 6B,H). We have summarized the telomere lengths of the different mutants in Supplementary Figure S3C.

To further examine the effects of the amino acid substitutions in the HEAT repeats, we sought to test them in full-length Rif1 and assess their effects at bulk telomeres. However, we found that most of these substitutions destabilized the full-length protein, with the exception of N867A W868A (Supplementary Figure S3D). This result was surprising given that these substitutions were stable in the context of the GBD Rif1 construct, suggesting that the smaller constructs fused to the GBD stabilized the Rif1 domains. Unfortunately, we cannot make conclusions from the long telomeres in these mutants (data not shown) because the long telomeres could be due to lower Rif1 protein levels.

Our data demonstrate that the conserved residues required for Rif1 telomere function cluster around the positively charged lysine residues, K437, K563, and K570. Our data do not support a model for the HEAT repeats simply mediating Rif1 DNA binding for potential telomere localization, since the lysine residues are important even when Rif1 is bound to the telomere, and both charged and uncharged residues compromise Rif1 function. Instead, we propose that this region may mediate a protein–protein interaction required for telomere length regulation.

## DISCUSSION

Rif1 has been known to regulate telomere length for over two decades, but we do not yet understand the mechanism of this regulation. We took a mutational approach to determine which regions of Rif1 are necessary for Rif1 function at the telomere. We localized different portions of Rif1 fused to the Gal4 DNA binding domain to a unique telomere at 1L. This approach enabled us to compare 1L telomere length to bulk telomere length to probe regions of Rif1 that function at the telomere. We identified a functional domain of Rif1 that, when affixed with an NLS, was sufficient to maintain telomere length similar to wild-type length at the unique telomere. This conserved region of HEAT repeats from aa 177–996 is comprised both of positively charged residues as well as other critical polar and nonpolar conserved residues, which are required for telomere length regulation. Previous work suggested this region may bind DNA (14); while we cannot exclude some possible regulatory role of DNA binding, our data indicate this region is not required for telomeric localization. Since residues other than positively charged amino acids had a major effect, we suggest that the Rif1 conserved HEAT repeats may provide a surface for protein-protein interactions, which regulates telomere length when Rif1 is at the telomere.

### Conserved NLS required for localization of Rif1 domains

We identified a highly conserved NLS in the Rif1 NTD, which is critical for telomere length regulation. Indeed, the functional role of the NTD can be replaced by an exogenous NLS and wild type telomere length was restored. This deletion analysis demonstrates that the primary role of the NTD is to localize Rif1 to the nucleus and that the PP1-binding site is not required for telomere length regulation. This finding supports our previous mutational analysis showing that substitutions in the PP1 binding region of Rif1 do not affect telomere length regulation (12). We also found that there is likely a region in the C-terminus that can partially compensate for loss of the N-terminal NLS. Identification of the NLS in the N-terminus is also of interest because in fruit flies and vertebrates a conserved NLS is present in the C-terminus (33,34). While Rif1 is conserved from yeast through humans, many functional domains of Rif1 appear to have been rearranged throughout evolution. For example, the N-terminal PP1 binding site is located in the C-terminus in mammalian Rif1 (15). Conservation of these regions suggests that, while there has been some rearrangement of domains, there may be evolutionary pressure to maintain these functional domains in Rif1 homologues.

### Rif1 HEAT repeats region is sufficient to regulate telomere length when localized to telomeres

The Rif1 region from aa 177–996, when tethered to the telomere, was able to maintain 1L telomeres similar to wild-type length. This region consists of conserved HEAT repeats, which typically promote protein–protein interactions (15,23). These HEAT repeats function independently of the most N-terminal region of aa 1–176 and the C-terminus of aa 997–1916 to maintain telomere length when bound to the telomere. This indicates that known functions in the N- and C-termini, namely PP1 binding, Dbf4 binding and Rap1 binding, are not required for Rif1 telomere function when Rif1 is tethered to the telomere.

The Rif1 HEAT repeats may mediate protein–protein interaction, not just DNA binding. Although Rif1 crystallizes with DNA as a dimer, it also crystallizes without DNA as a monomer (PDB: 5NVR) (14). The authors proposed that DNA binding may add an additional mechanism to localize Rif1 to telomeres, even though the DNA binding was weak. Given that Rif1 also crystallized as a monomer without DNA (PDB: 5NW5) (14), perhaps dimerization and DNA binding are not important *in* vivo. Here we confirmed that the lysine residues implicated in DNA binding have functional importance in regulating telomere length, but their function likely extends beyond telomere localization, as they are required for telomere length regulation even when tethered to the telomere (Figure 5). We found that several conserved residues proximal to these positively charged lysine residues, which were not predicted to interact with DNA, were also critical for telomere length regulation. On the other hand, conserved residues distal to this region did not affect telomere length (Figure 6C,D,E vs Figure 6F). The small surface around these lysine residues (K437, K563, K570), between M436 and Y577, which has the greatest impact on telomere function, is also the most conserved HEAT repeat from yeast to humans (15). While we cannot rule out that the HEAT repeats may interact with DNA to provide some function other than telomere localization, we propose this conserved region may recruit other proteins to regulate telomere length.

### Rif1 HEAT repeats have partial function when not tethered to the telomere

While Rif1 HEAT repeat constructs restored wild-type telomere length when localized by GBD to the 1L telomere, we found that they also had a small effect on bulk telomere length (Figures 1D, 3E and 4D). This finding was surprising as Rif1_1–1322_ and Rif1-NLS_177–996_ both lack Rap1 binding and, therefore, cannot localize to the telomere (13,35,36). For Rif1_1–1322_, this partial effect has been previously reported (12,13). These data suggest that these constructs can function in telomere length regulation by an unknown mechanism that is independent of Rap1 binding. We suggest that localizing Rif1 to the telomere, through either Rap1 binding or GBD, promotes a high local concentration of Rif1; however, Rif1 can still partially perform its function of regulating telomere length when not telomere bound. Perhaps there is simply a lower local concentration when Rif1 is not tethered to the telomere, leading to the partial length regulation that is seen. This conclusion is supported by longstanding evidence that recruiting more Rif1 to the telomere, by adding Rap1 binding sites or by directly tethering full-length Rif1, leads to progressively shorter telomeres (37,38).

Despite the conserved role of Rif1 homologues in origin firing, its role as a telomere regulator has only been characterized in yeasts (39–41). Moreover, localization to telomeres has only been shown in yeast, although through different mechanisms (29). We have shown that even heterologous tethering of Rif1 to a telomere negatively regulates telomere length. This finding suggests the possibility that, if Rif1 were present at higher concentration at telomeres in other organisms, it might exert a negative effect on telomere elongation.

### Evolutionarily conserved HEAT repeats are important in both NHEJ and telomere length

The Rif1 HEAT repeats region, which we found is critical for carrying out telomere length regulation, is also implicated in promoting NHEJ in both yeast and mammals. This region, which contains the positively charged lysines (K437, K563, K570), is required to promote NHEJ in yeast, and this effect does not require binding to Rap1 (14). The importance of the positively charged residues in the HEAT repeats may indicate that Rif1 is binding to a negatively charged protein domain or, possibly, a phosphorylated protein. In mammalian cells, Rif1 HEAT repeats are specifically required for Rif1 localization at double-strand breaks (18). The ATM-dependent phosphorylation of 53BP1 recruits Rif1 to sites of DNA damage to block end resection and promote NHEJ (16–19,41,42). This suggests a possible role for the 53BP1 yeast ortholog Rad9, which is similarly phosphorylated at S/T-Q residues by Tel1/Mec1 (43,44). However, Rad9 does not have a telomere length effect on its own, so perhaps the HEAT repeats bind to and regulate the activity of another protein to promote both NHEJ and telomere length regulation. Additionally, while Tel1 functions in the same genetic pathway as Rif1 (26), its role in telomere length regulation is also not fully understood. Our data and others’ (26) indicate that Tel1 phosphorylation of Rif1 is not the mechanism by which Tel1 regulates telomere length (Figure 4). Understanding the roles of Tel1 phosphorylation and Rif1 in both telomere length regulation and NHEJ will allow more complete understanding of Rif1 function.

We have dissected the domains of Rif1 that function to regulate telomere length. We found that the Rif1 HEAT repeats region between M436 and Y577 is primarily responsible for Rif1 telomere function, and that the N- and C-termini of Rif1 are not required for Rif1 function in telomere length regulation. While we cannot rule out some role for DNA binding of this region, our data are most consistent with the Rif1 HEAT repeats interacting with a protein partner to carry out its telomere function. The partial function of Rif1 when not bound to the telomere suggests that the local concentration of Rif1 is important for its function in telomere length regulation. These results suggest several exciting questions to understand Rif1 function in telomere length regulation: are these functions conserved or yeast specific?; which protein partners are interacting with Rif1 to carry out these functions?; and is the conserved role of Rif1 in NHEJ commandeered in yeast for telomere regulation?

## Supplementary Material

gkab206_Supplemental_File

## Notes

Present address: Ariel D. Swett, Sandra and Edward Meyer Cancer Center, Weill Cornell Medicine, New York, NY 10065, USA.
