# Supplementary material for: Rif1 regulates telomere length through conserved HEAT repeats
Source: Nucleic Acids Res. 2021 Mar 27;49(7):3967–80. doi: 10.1093/nar/gkab206 (PMC8053089; doi:10.1093/nar/gkab206)
Supplement: gkab206_Supplemental_File [file gkab206_supplemental_file.pdf]

Figure Legends for Supplementary Figures

Supplementary Figure S1: ***N-terminal NLS is important in full length Rif1***

A. Western blot showing Rif1 (anti-FLAG antibody) and Pgk1 (anti-Pgk1 antibody, control) protein levels of indicated strains. B. Southern blot hybridized with a Y' probe to visualize "bulk" XhoI restriction fragments of indicated strains (See Materials and Methods).

Supplementary Figure S2: ***Structure-based sequence alignment***

A. Workflow of structure-based sequence alignment (see Materials and Methods). B. Phylogenetic tree of the Multiple Sequence Alignment (MSA) generated using ConSurf. C. Conservation graph generated using GraphPad Prism of sequence alignment with position in Rif1 protein on the X-axis. The height of the bar indicates ConSurf Grade, with a higher bar having higher conservation. Purple indicates high conservation, and green indicates low conservation. Rif1 domain map is depicted to scale above the bar graph, with the newly defined NLS added, and Rif1 aa 177-196 are depicted with a blue dotted line (RBM: Rap1 binding motif; CTD: Carboxyl-terminal domain; Dbf4 binding overlaps CTD). D. MSA mapped onto the Rif1 crystal structure (PDB: 5NW5, showing one Rif1 monomer and DNA) visualized using PyMOL. Purple indicates high conservation, and green indicates low conservation.

Supplementary Figure S3: ***Protein levels for strains mutated in conserved HEAT repeats***

A. Western blot showing Rif1 (anti-FLAG antibody) and Pgk1 (anti-Pgk1 antibody, control) protein levels of indicated strains, related to Figure 6A,B. B. Western blot showing Rif1 (anti-FLAG antibody) and Pgk1 (anti-Pgk1 antibody, control) protein levels of indicated strains, related to Figure 6F,G. C. Conserved residues with corresponding distance from lysine cluster and 1L telomere length, related to Figure 6A. D. Western blot showing Rif1 (anti-FLAG antibody) and Pgk1 (anti-Pgk1 antibody, control) protein levels of indicated strains.

Supplementary Figure S4: Mutation of ***Rif1 palmitoylation sites does not affect telomere length***

A. Western blot showing Rif1 (anti-FLAG antibody) and Pgk1 (anti-Pgk1 antibody, control) protein levels of indicated strains. B. Southern blot hybridized with a Y' probe to show bulk telomeres for the indicated strains (See Materials and Methods).

Supplementary Figure S1

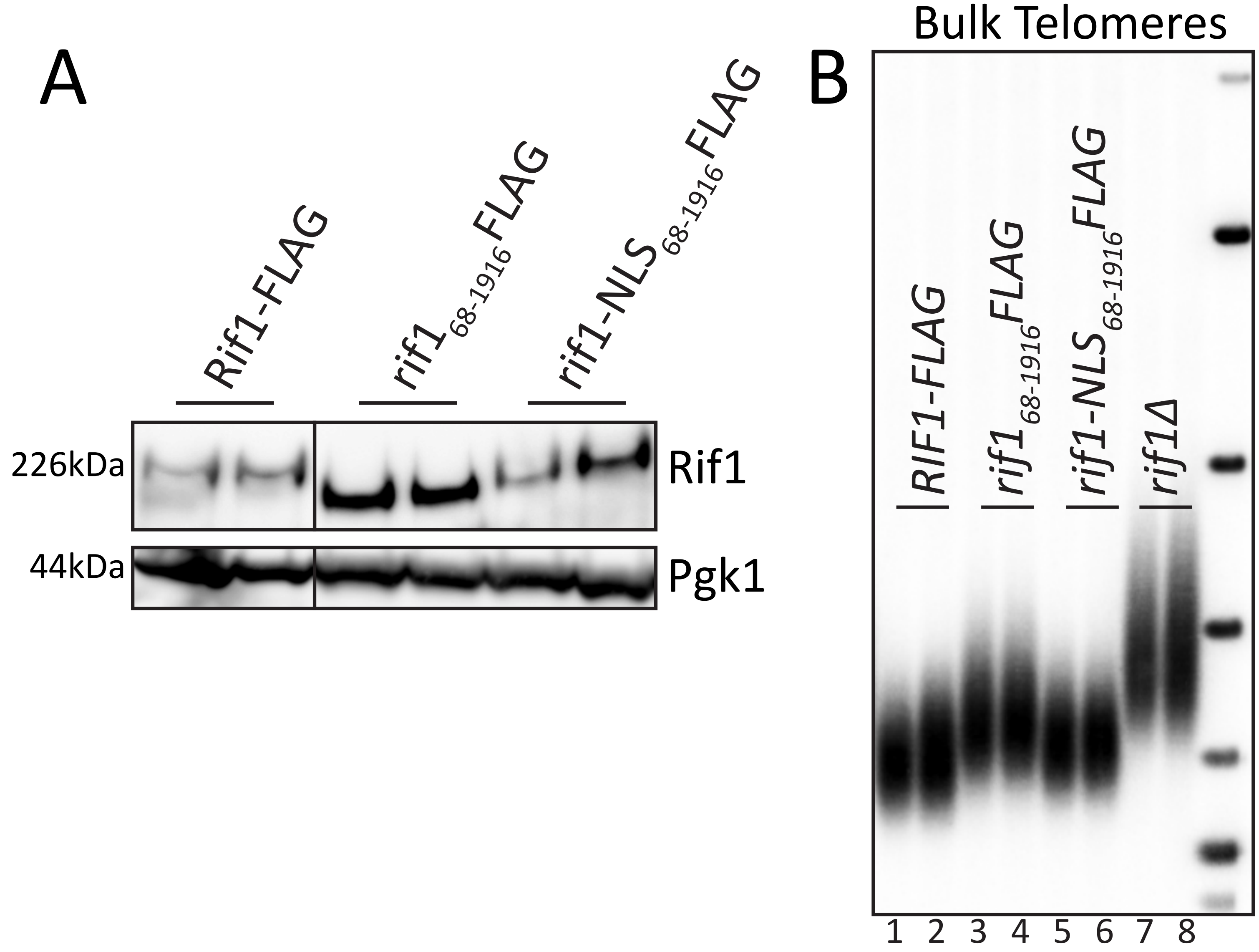

Supplementary Figure S2

A

Compile sequences of 12 yeast Rif1 orthologs

↓

Generate Multiple Sequence Alignment (MSA) using Clustal Omega

↓

Generate interactive phylogenetic tree of MSA using ConSurf

↓

Map MSA onto crystal structure using ConSurf

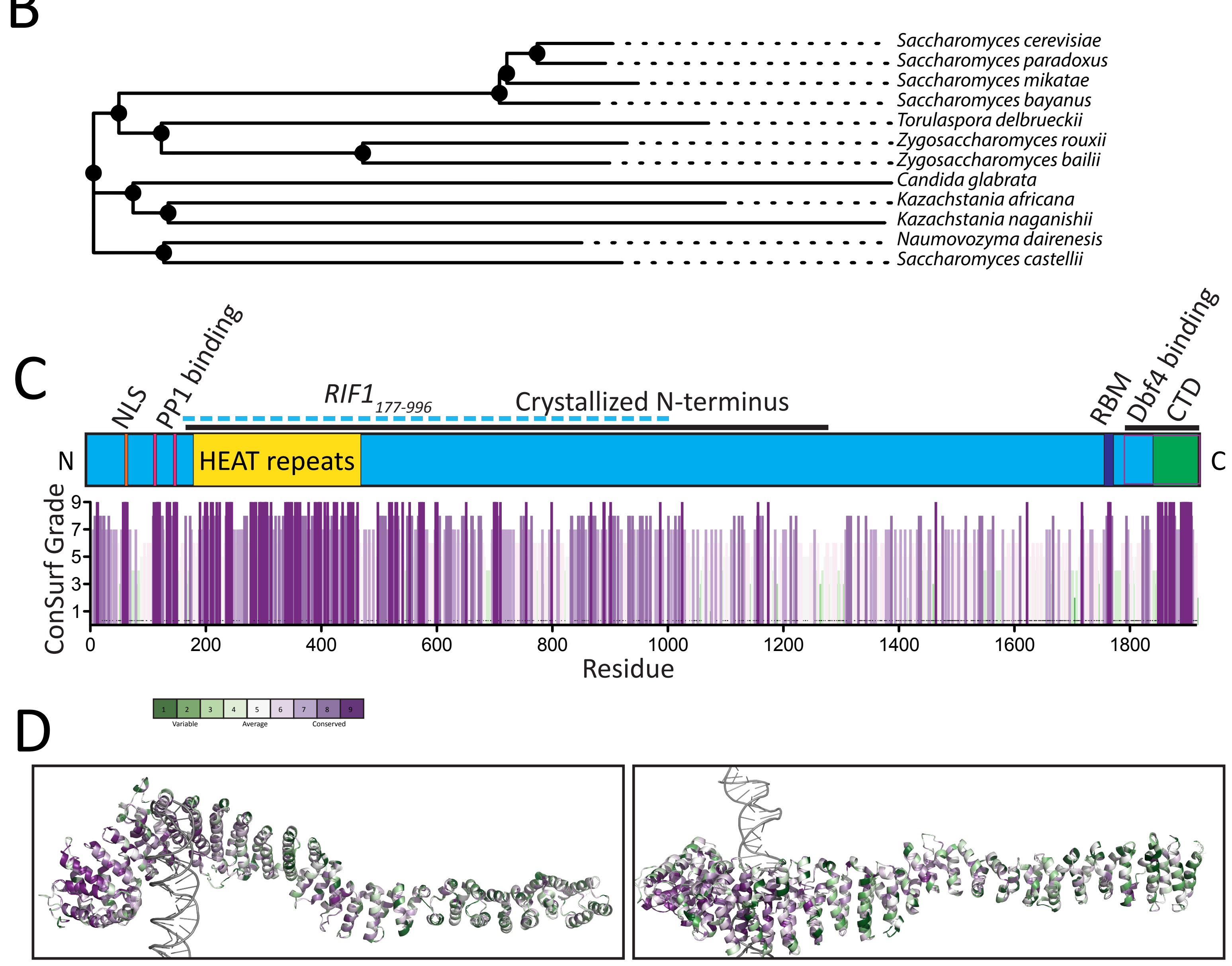

Supplementary Figure S3

A

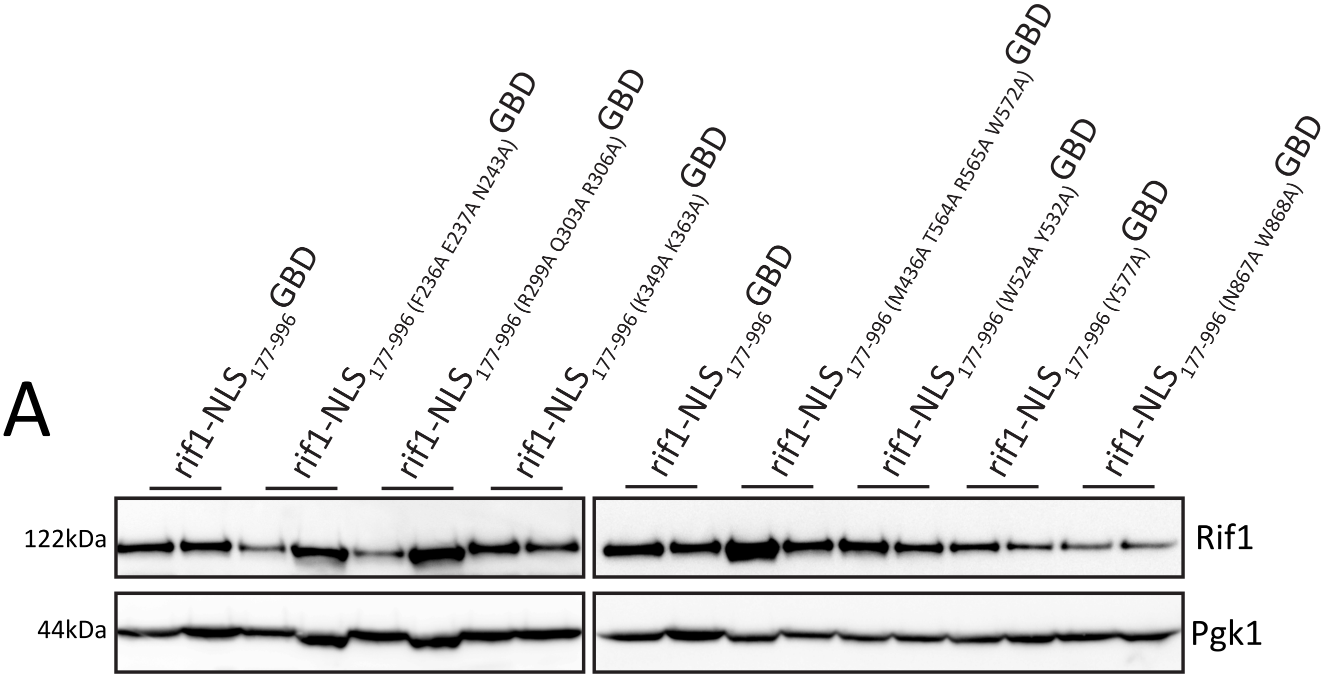

B

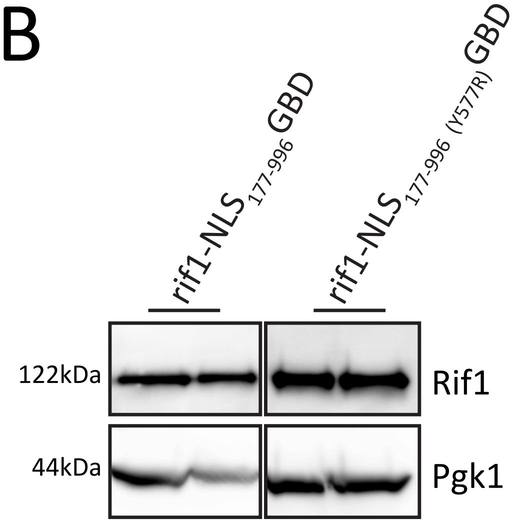

C

Residues Interrogated	Distance from Lysine cluster	1L Telomere length
F236A E237A N243A	194	Wild type
R299A Q303A R306A	131	Wild type
K349A K363A	74	Wild type
M436A T564A R565A W572A	0	Long
K437A K563A K570A	***0***	***Long***
W524A Y532A	0	Long
Y577A	7	Long
N867A W868A	297	Wild type

D

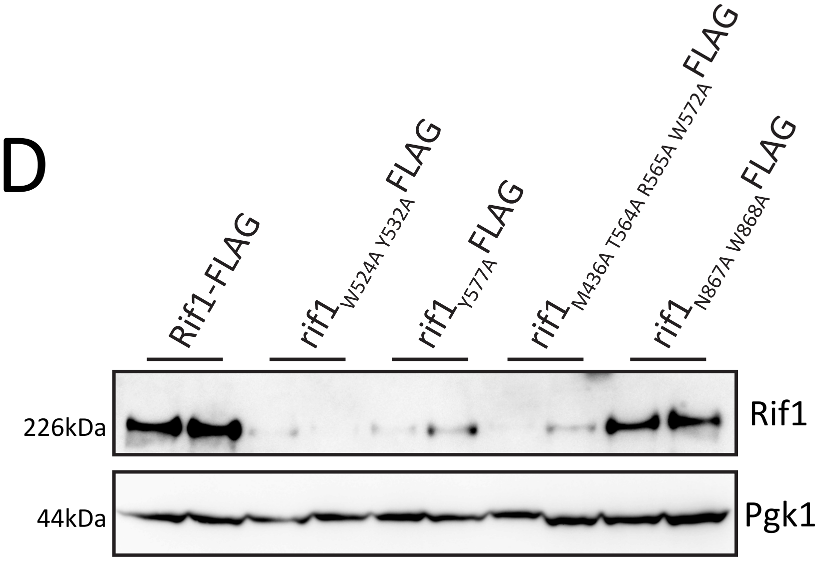

Supplementary Figure S4

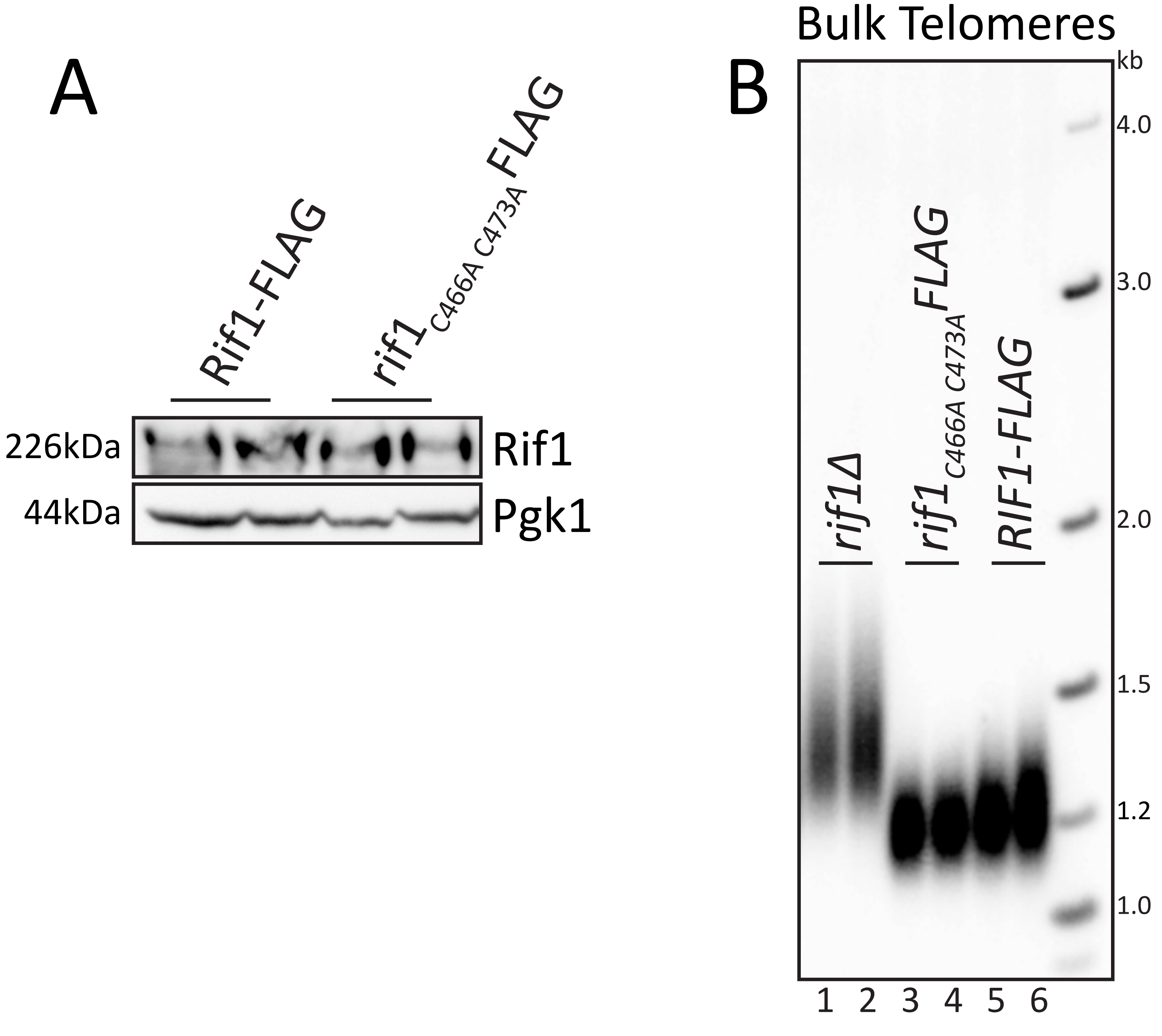

Figure Legend for Supplementary Table

Supplementary Table 1: ***Strain list, plasmid list, and cloning specifics and reagents***

Supplementary Table 1

Strain ID	Genotype	Source	Construct for cloning	Primer 1	Primer 2	Additional information
SSy161	MATa ade2-1 ura3-1 his3-11,15 trp1-1 leu2-3,112 can1-100 bar1::hisG	N/A				
CVy61	SSy161 + TRP1::BrdU–Inc	https://doi.org/10.1002/yea.1406				
CALy11	CVy61 + rif1Δ::kanmx	https://doi.org/10.7554/eLife.58066				
CALy320	CALy117 + rif11-1322-6xFLAG-KANMX	This study	pAS35	AS101	CS201	Extend homology by PCR with CS315, AS100
JHUy783	tel1Δ::HYGR	Greider Lab				
CALy117, 118	CVy61 + 1L 5XUAS landing pad	This study	pCS33	CS155	CS218	Digest PCR product I-Sce1 DpnI
CALy202, 203	CALy117 + rif1Δ::KANMX	This study	gDNA (CALy11)	OCC23	OCC84	
CALy286, 287	CALy117 + rif11-1322GBD-6xFLAG-KANMX	This study	pCS172	RW432	OCC84	
CALy290, 291	CALy117 + rif11-1322(K437E K563E K570E) GBD-6xFLAG-KANMX	This study	pCS171	RW428	OCC84	
CALy328, 329	CALy117 + rif11-1322-6xFLAG-KANMX	This study	gDNA (CALy320)	RW431	CS323	
CALy375, 376	CALy117 + rif1177-1322GBD-6xFLAG-KANMX	This study	pCS184	OCC23	OCC84	
CALy408, 409	CALy117 + rif1-cmycNLS177-1322GBD-6xFLAG-KANMX	This study	pCS189	OCC23	OCC84	
CALy428, 429	CALy117 + rif11-996GBD-6xFLAG-KANMX	This study	pCS193	RW430	CS323	
CALy432, 439	CALy117 + rif1-cmycNLS177-996GBD-6xFLAG-KANMX	This study	pCS193	OCC23	CS323	
CALy442, 443	CALy117 + RIF1-6xFLAG-KANMX	This study	pCS177	AS100	CS383	
CALy454, 455	CALy117 + rif1-cmycNLS177-996 (K437E K563E K570E)GBD-6xFLAG-KANMX	This study	pCS195	OCC23	OCC84	
CALy460, 461	CALy117 + rif1-cmycNLS177-996 (M436A T564A R565A W572A)GBD-6xFLAG-KANMX	This study	pCS198	OCC23	OCC84	
CALy462, 463	CALy117 + rif1-cmycNLS177-996 (K437R K563R K570R)GBD-6xFLAG-KANMX	This study	pCS196	OCC23	OCC84	
CALy465, 465	CALy117 + rif1-cmycNLS177-996 (T504E S584E T775E S824E)GBD-6xFLAG-KANMX	This study	pCS210	OCC23	OCC84	
CALy472, 473	CALy117 + tel1Δ::HYGR	This study	gDNA (JHUy783)	OCC25	OCC80	
CALy478, 479	CALy117 + rif1-cmycNLS177-996 (T504A S584A T775A S824A)GBD-6xFLAG-KANMX	This study	pCS209	OCC23	OCC84	
CALy480, 481	CALy117 + rif1-cmycNLS177-996 (K349A K363A)GBD-6xFLAG-KANMX	This study	pCS207	OCC23	OCC84	
CALy486, 487	CALy472 + rif1-cmycNLS177-996GBD-6xFLAG-KANMX	This study	pCS193	OCC23	CS323	
CALy488, 489	CALy472 + rif1-cmycNLS177-996 (T504A S584A T775A S824A)GBD-6xFLAG-KANMX	This study	pCS209	OCC23	OCC84	
CALy490, 491	CALy472 + rif1-cmycNLS177-996 (T504E S584E T775E S824E)GBD-6xFLAG-KANMX	This study	pCS210	OCC23	OCC84	
CALy498, 499	CALy117 + rif1-cmycNLS177-996 (N867A W868A)GBD-6xFLAG-KANMX	This study	pCS211	OCC23	OCC84	
CALy503, 539	CALy117 + rif1-cmycNLS177-996 (F236A E237A N243A)GBD-6xFLAG-KANMX	This study	pCS214	OCC23	OCC84	
CALy505, 546	CALy117 + rif1-cmycNLS177-996 (R299A Q303A R306A)GBD-6xFLAG-KANMX	This study	pCS215	OCC23	OCC84	
CALy506, 507	CALy117 + rif1-cmycNLS177-996 (W524A Y532A)GBD-6xFLAG-KANMX	This study	pCS216	OCC23	OCC84	
CALy508, 509	CALy117 + rif1-cmycNLS177-996 (Y577A)GBD-6xFLAG-KANMX	This study	pCS217	OCC23	OCC84	
CALy520, 521	CALy117 + rif1-cmycNLS177-996 (K437A K563A K570A) GBD-6xFLAG-KANMX	This study	pCS222	OCC23	OCC84	
CALy530, 531	CALy117 + rif1-cmycNLS177-996 (Y577R)GBD-6xFLAG-KANMX	This study	pCS230	OCC23	OCC84	
CALy532,533	CALy117 + rif1-68-1916-6xFLAG-KANMX	This study	pCS227	Kpn1	NotI	Digested plasmid, gel extracted top band for transformation
CALy534,535	CALy117 + rif1-cmyc-68-1916-6xFLAG-KANMX	This study	pCS226	Kpn1	NotI	Digested plasmid, gel extracted top band for transformation
CALy547,548	CALy117 + rif1-W524A Y532A-6xFLAG-KANMX	This study	pCS234	RW428	CS323	
CALy549,550	CALy117 + rif1-Y577A-6xFLAG-KANMX	This study	pCS233	RW428 or CS509	CS323	
CALy551,552	CALy117 + rif1-M436A T564A R565A W572A-6xFLAG-KANMX	This study	pCS235	RW428	CS323	
CALy553,554	CALy117 + rif1-C466A C473A-6xFLAG-KANMX	This study	pCS237	RW428	CS323	
CALy557,558	CALy117 + rif1-N867A W868A-6xFLAG-KANMX	This study	pCS239	RW429	CS323	

Figure 1

CALy117, 118

CALy202, 203
CALy328, 329
CALy286, 287
CALy442, 443

Figure 2

CALy117, 118
CALy202, 203
CALy328, 329
CALy286, 287
CALy375, 376
CALy408, 409

Figure 3

CALy117, 118
CALy328, 329
CALy286, 287
CALy408, 409
CALy428, 429
CALy432, 439
CALy202, 203

Figure 4

CALy117, 118

CALy328, 329
CALy202, 203
CALy472, 473
CALy432, 439
CALy486, 487
CALy478, 479
CALy488, 489
CALy465, 465
CALy490, 491

Figure 5

CALy117, 118
CALy328, 329
CALy286, 287
CALy290, 291
CALy432, 439
CALy454, 455
CALy462, 463
CALy520, 521

Figure 6

CALy117, 118

CALy503, 539
CALy505, 546
CALy480, 481
CALy460, 461
CALy506, 507
CALy508, 509
CALy498, 499
CALy432, 439
CALy530, 531

Supplementary Figure S1

CALy442, 443
CALy532,533
CALy534,535
CALy202, 203

Supplementary Figure S3

CALy117, 118

CALy503, 539
CALy505, 546
CALy480, 481
CALy460, 461
CALy506, 507
CALy508, 509
CALy498, 499
CALy432, 439
CALy530, 531
CALy442,443
CALy547,548
CALy549,550
CALy551,552
CALy557,558

Supplementary Figure S4

CALy442, 443
CALy553,554
CALy202, 203

PRIMERS	SEQUENCE	SOURCE
AS100	GCGAATTTTGAAAGATTGTGCGAATTATTGGGTTATGATAACGTTTTCCC	This study
AS101	TATCCAGGTTCCGGCTACGCAGGGAATGAAAGAGCCCGGGATACATTATACGAAGTTATG	This study
CS155	GATGGATATCTGCAGAATTCGCCCTTATTACCCTGTTATCCCTACACAC	This study
CS201	AGTGAGCGCGCGTAATACGACTCACTATAGGGCGAATTGG	This study
CS218	CTCGAGCCGAACGTTCTTACTCTCATATCCCTACTTAGGTTCAGTTAGCCGTACAACTGTGGGAATACTCAGGT	This study
CS315	CAAACTTACAGAGAAAAGTGGATTCTCAAGATATCCAGGTTCCGGCTACG	This study
CS323	GTGCGAATTATTGGGTTATGATAACG	This study
CS383	AAGAAACTTGAGAATTGAATTATTAGATTTTATGATGAGGCTCGAATATTACTCAAACAG	This study
CS509	CCAAATAGTTCGATTCTTCAGTGC	This study
OCC23	TTTTCAGTTCTTTGTGTTTTTCCTC	This study
OCC25	CACATGATATTATGAGCGTGATAGG	This study
OCC80	ATCTACGTCGATTTCTTTCATTTTG	This study
OCC84	GATAAGGATGCCAATATTAATGACG	This study
RW428	TGTTAACGAGGTTGATCAGAAATTCAA	This study
RW429	AGCAATGGATTTGTGGCTTTCA	This study
RW430	TTTTTGGTGCTGTTACTTCATCGT	This study
RW431	ACAAAATGCGTCTGATAGCGAGT	This study
RW432	TCATCAAGGGGACTCAAGTAAGGA	This study

PLASMID	DESCRIPTION	SOURCE
pCR2.1	Thermo Fisher Scientific	Thermo: K202020
pAS35	rif11-1322 (PP1BS (K114A V116A F118A I147A L148A R149A) - GBD-6xFLAG-KANMX	This study
pCS33	pCR2.1 + 5XUAS landing pad	This study
pCS171	rif11-1322 (PP1BS (K114A V116A F118A I147A L148A R149A) - HOOK (K437E K563E K570E) GBD-6xFLAG-KANMX	This study
pCS172	rif11-1322 (PP1BS (K114A V116A F118A I147A L148A R149A) - HOOK (K437E K563E K570E) - LOOP (K691E K692E) GBD-6xFLAG-KANMX	This study
pCS177	pCR2.1 + rif11892-1916-6xFLAG-KANMX	This study
pCS184	rif1177-1322GBD-6xFLAG-KANMX	This study
pCS189	rif1-cmycNLS177-1322GBD-6xFLAG-KANMX	This study
pCS193	rif1-cmycNLS177-996GBD-6xFLAG-KANMX	This study
pCS195	rif1-cmycNLS177-996 (K437E K563E K570E) GBD-6xFLAG-KANMX	This study
pCS196	rif1-cmycNLS177-996 (K437R K563R K570R) GBD-6xFLAG-KANMX	This study
pCS198	rif1-cmycNLS177-996 (M436A T564A R565A W572A) GBD-6xFLAG-KANMX	This study
pCS206	pCR2.1 + 1L Telomere probe	This study
pCS207	rif1-cmycNLS177-996 (K349A K363A) GBD-6xFLAG-KANMX	This study
pCS209	rif1-cmycNLS177-996 (T504A S584A T775A S824A) GBD-6xFLAG-KANMX	This study
pCS210	rif1-cmycNLS177-996 (T504E S584E T775E S824E) GBD-6xFLAG-KANMX	This study
pCS211	rif1-cmycNLS177-996 (N867A W868A) GBD-6xFLAG-KANMX	This study
pCS214	rif1-cmycNLS177- 996 ((F236A E237A N243A) GBD-6xFLAG-KANMX	This study
pCS215	rif1-cmycNLS177- 996 (R299A Q303A R306A) GBD-6xFLAG-KANMX	This study
pCS216	rif1-cmycNLS177- 996 (W524A Y532A) GBD-6xFLAG-KANMX	This study
pCS217	rif1-cmycNLS177- 996 (Y577A) GBD-6xFLAG-KANMX	This study
pCS222	rif1-cmycNLS177-996 (K437A K563A K570A) GBD-6xFLAG-KANMX	This study
pCS227	rif1-68-1916-6xFLAG-KANMX	This study
pCS226	rif1-cmyc-68-1916-6xFLAG-KANMX	This study
pCS230	rif1-cmycNLS177- 996 (Y577R) GBD-6xFLAG-KANMX	This study
pCS234	rif1-W524A Y532A-6xFLAG-KANMX	This study
pCS233	rif1-Y577A-6xFLAG-KANMX	This study
pCS235	rif1-M436A T564A R565A W572A-6xFLAG-KANMX	This study
pCS237	rif1-C466A C473A-6xFLAG-KANMX	This study
pCS239	rif1-N867A W868A-6xFLAG-KANMX	This study

Cells and enzymes for cloning and Southern digest	SOURCE	SOURCE
Gibson Assembly master mix	New England Biolabs	NEB: E2611L
High Prep PCR Clean Up beads	MagBio	MagBio: AC-60050
NEB5a	New England Biolabs	NEB: C2987H
TOP10	Thermo Fisher Scientific	Thermo: C404010
DH5a	Thermo Fisher Scientific	Thermo: 18265017
I-Sce1	New England Biolabs	NEB: R0694L
DpnI	New England Biolabs	NEB: R0176L
XhoI	New England Biolabs	NEB: R0146L
PvuII-HF	New England Biolabs	NEB: R3151L
Kpn1	New England Biolabs	NEB: R0142S
NotI	New England Biolabs	NEB: R0189S

1L TELOMERE PROBE FOR SOUTHERN- PCR from pCS206

CS414	ATCACCAGCAACATGTTTGG	This study
CS432	GCAAATTAAAGCCTTCGAGCG	This study

Y' PROBE FOR SOUTHERN- PCR from gDNA

YPrimeFWD	CGCGAATTCGCCCTACAGCACTTCTACATAGC	doi.org/10.1534/genetics.115.177899
YPrimeREV	CGAGAATTCCAGCGTTTGCGTTCCATGACG	doi.org/10.1534/genetics.115.177899

YEAST MEDIA SUPPLEMENTS

NAM (Nicotinamide)	5mM final to plates	Sigma: 72340-100G
G418 (Geneticin)	200µg/ml final to plates	Life Tech: 11811031
Hygromycin	200µg/ml final to plates	Corning: 30-240-CR

Figure Legend for Supplementary File

Supplementary File 1: ***Multiple Sequence Alignment (MSA) of Rif1***

MSA of Rif1 generated as described in Materials and Methods and visualized using SnapGene software.

Consensus
1. Saccharomyces_cerevisiae
2. Kazachstania_naganishii
3. Candida_glabrata
4. Saccharomyces_castellii
5. Naumovozyma_dairenensis
6. Kazachstania_africana
7. Torulaspora_delbrueckii
8. Saccharomyces_mikatae
9. Saccharomyces_bayanus
0. Saccharomyces_paradoxus
1. Zygoaccharomyces_bailii
2. Zygosaccharomyces_rouxii

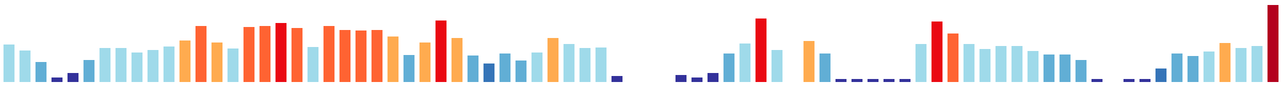

```
    MSX--XXXXXXXKXXRAIDXLDXHIXXRSXXXXXSXXX------XXXW-XX-----XLPXXXXXXXX----XXXXXXXS

1   -----MSKDFSDKKKHTIDRIDQHILRRSQHDNYSNGS-------SPWM-KT-----NLPP-PSPQAH----MHIQSDLS   57
2   MTEHSTERSSNRMINRTLEMLEKAGSKKNT---FAN--------KYPWS-SGNFKRNNAPIPEHTD-------SRDYSTS   61
3   MSD---GSADRKGKMRALDDILSKHLRGKNSDKTTPTRYGHKSVELESILRKDNIVSSPLESRDHQESQR-INNPHSNTAS   76
4   MSDTPTKNTSNKKSDRAIDLLNKQLKLK-----NSRSSP---IIPLQWF-NS-----QVSQQESS--------FNPNELS   58
5   MSDSSKESSGPRKNDRAITLLDKHINQKSKMKSTSNSSP---IVLTQWF-NS-----QLPAPPSTSPVSVQTHKAANDVS   71
6   MSDESHKDASPKKNMKALAMIDKHISQRTLQKKNLSPS-------STWV-KP-SMSNQLSAQPQQNAAY---WNSANNTS   68
7   MSTKPDGDMHREKKMRAIDMLNHFINKRSQAKAKSVSPT-----ARNWK-NS-----RLSPPNTEEPN-----EDANNVS   64
8   -----MSKDFSDKKKHTIDRIDQHILRRSQHDVNPNGS-------SPWM-KT-----KLPPLPFPQAH----MHIQSDLS   58
9   -----MSNDFSDKKKHTIDRIDQHILRRSQQDAHSSGN-------SPWM-KA-----KLPPPPSPQAH----MHMQSDLS   58
10  -----MSKDFSDKKKHTIDRIDQHILRRSQHDAHSNGS-------SPWM-KV-----NLPPPPSPQAQ----MHIRSDLS   58
11  MSS--PPGQSTEKRMRAIDRLDQHMIKRSKSNNRASSP----VPFLSWI-TQ-----KLPPPSSLLGQ----RDAPTQTS   64
12  MSS--SSNQSSEKRMRAIDRLDQHMMKRSMKNSKSSSPS---GPFLPWL-SH-----KLPSNSQSDSNG---SQNHEISS   66
```

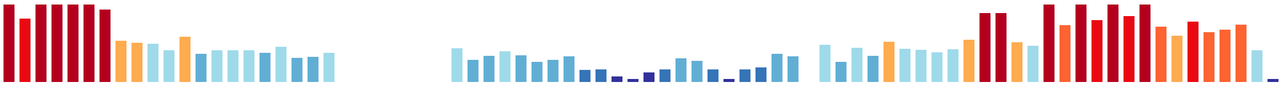

```
    PTPKRRKXXXXSXXXXXXXXX-------XXXXXXXXXX--XXXXX-XXXX-EXXXSXXXXXSPXXKSVAFSDRXESSPX-

1   PTPKRRKLASSSDCENKQFDL-------SAINKNLYPEDTGSRLM-QSLP-ELSASNSDNVSPVTKSVAFSDRIESSPI-   127
2   PTPKRRKMATPQSADKTTVQA-------NSNSLDNLK----ISL---VFP-KDSERPLSSSSPCQKSVSFSDRIEVESSS   126
3   PTPKRRKLNTQAISSDNKTKQVLSIGELSHPDMQSTPA--RSRIMKKDIGDQNMSSSEIYSPSVNKSVSFLDESNKNSE-   153
4   PTPKRRKLNNKRPATEKSFKK-------NAINLEETPILMAPKSISDTR----FDDPISNSSPIRKAVAFSDKVESSPTQ   127
5   PTPKRRRVNNMDQTSSTVPHL----------DLRRF------ELNNDTRL-ERSTSESNSASPIRKTVAFSDKIEIESSP   134
6   PTPKRRKITSSSEATLPEGSK-------NFTVLNKAFE----------VV-GQGPSEDILSSPGQKSVAFSDRIDSSPE-   129
7   PTPKRRKFAQSSPVRKPEEPV-------LSTDV-------QLK----LPA-TESLNDKAEGSPGTKSVAFSDKLDSSPT-   124
8   PTPKRRKLAFSSNYDNKELDL-------SANYKNLYSEDEGSKLV-QKLP-ELPPSNSDSVSPVTKSVAFSDRIESSPV-   128
9   PTPKRRKLLSPSISENDQLDL-------SKSHEHSSLEDVNNKLM-QSLP-EISASNSNNVSPVTKSVAFSDRIESSPI-   128
10  PTPKRRKLASSSNCENKQSDL-------STTNTNSYSEDAGNRLM-QSLP-ELSASNSDNVSPVTKSVAFSDRLESSPL-   128
11  PSPKRRKTRSLSPSKIAEP-R-------NRHAG-TF------------AD-ENRCGTGANSSPVKKSVAFSDKLESSPT-   121
12  PSPKRRKTASLSPIRNVESTI-------DGPSN-TF------------PI-EARVVTSVQSSPIKKTVAFSDRVESSPT-   124
```

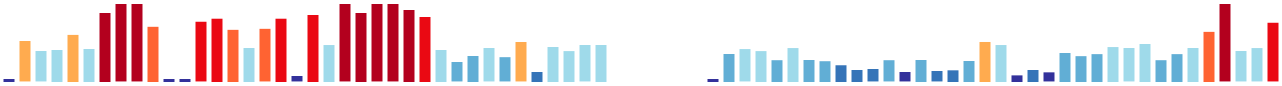

```
-XXXPXSSPR--PSXXSK-PXKSILRNXXXXXXXXXSD-------XXXXKXXXXXXXLXXXXIXXXXXXXXXXXXXPXXL

1   -YRIPGSSPK--PSPSSK-PGKSILRNRLPSVR-TVSD-------LSYNKLQYTQHKLHNGNIFT-SPYKET-RVNPRAL  193
2   AQTNSAASPR--PSSTSKLRSKPILKLNRVSLQKGDIV--------TE-------Q-----RL--TFSARTTFSLNPMEL  182
3   -STSSNSSPR--HSPTSK-PKKSILRNSSSPEHNKQRM------NDKYGKPIAANV-----HL-SRSENHSKNQYSPDNL  217
4   QSPMPDSSPRRPPSSATKPPGKSILRNPDFITNRTKSD------------------LLSNKDK--LVDSMSPLQLDPGNI  187
5   TQTLPESSPRHPPSSDRKPPSKSILRNSKTPSSPVKYNTIKASSDVTYSKTVS--NNLAASNA--GVATNDFLHADPRKV  210
6   -RITAKSSPT--PSSTSKPPVKSILRNPGATQD-TDEE-------LNHEKSQR-------DSI--VPIDNDSSETNPNNI  189
7   -QRTMNSSPR--QTPASA-PSKSILRNPIEFQK-NVSL-------MADP-AGKPKSKLA-GR-G-GQVNDTSDDIDPTSL  188
8   -HHVPGSSPK--PSPSSR-PGKSILRNKMSSAR-TVSD-------LSYNKLQHPEHRLHNGNILT-SPYKETIVINPRTL  195
9   -HHVPGSSPK--PSPSSK-PGKSILRNRMPPMR-TVSD-------LSYNKLQHSQHQLHNGNIFT-SPYKESTGVDPRTL  195
10  -HHVPGSSPK--PSPSSK-PGKSILRNRMPSVR-TVSD-------LSYNKLQYTQHKLHNGNIFT-SPYKETIGLNPHSL  195
11  -QKSPRSSPR--PTSVSK-PSKSILRNNADTFRKSTGD-------LTAKRSELMLT----SHIGSARLENNTSPTDPHSL  186
12  -QQTPRSSPR--PSSLQK-PVKSILRNT-DALRKCVSD-------ISFKRPPFLNQ-----NIDR-NKTDRTAGIDPHRL  186
```

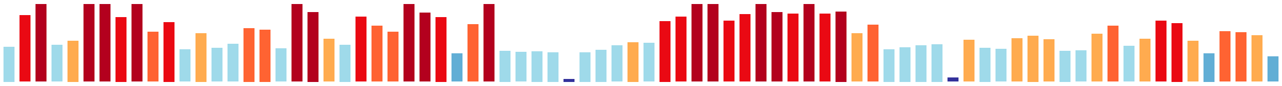

```
XYWVSGEIHSLXDXXNVXEFKXIIEGGLXILXXXX-XXXVXXRFEXYATFNNIIPXXXX-KXXXEXXXXKXNVLIXNXEX

1   EYWVSGEIHGLVDNESVSEFKEIIEGGLGILRQES-EDYVARRFEVYATFNNIIPILTT-KNVNEVD-QKFNILIVNIES  270
2   AYWRSGEIHNMTDIKNINELKNIFEGGLSLLAVSE-PEHVSKRFEIYATFNAILERQFGDNTPTGMEDKVCSVLCAHMEE  261
3   QFWGNGEIHSLSSNNSVAEFEKLFLGALKILMQSKNPEYGAKKFEVHATINNIMPSFSS-TTSNQFIEKAAHIVKDNLKI  296
4   EYWVSGEIHSLFNNENVKEFEGIIIGGIHILQLENVENSSNRRFEIYATFNNIIPILPTDKDIDPITENKVKVIVNNLPE  267
5   EYWADGEIHSLLYPNNIKEFRDIIEGGISFLNM------GLKKFEIFATFNNIIPIIPNGFNATDVIDKKIGIIVENLDN  284
6   SYWTGGEIHSLKDHNNLAEFKQVIEGGLYVLSSNS-DPHNSRRFEIYATFNNLCRGLSASDHVSETNEKKINILFQKLET  268
7   AFWEFGEVHKMLDSSNFKEFQKIIEGGLEILANNE-RSHVARRFEVYASFNNIIPPNNL-KYHSELIEKKFDILIHNLKK  266
8   NYWVSGEIHGLIDNESISEFKEIIEGGLNILRKES-EEYVAKRFEVYATFNNIIPILTT-KNVNEVD-QKFNVLIVNIEA  272
9   DYWVSGEIHSLIDNESVSEFKDIIEGGLGILRQES-EEYVARRFEVYATFNNIIPILTT-KNVNEVD-QKFNVLIVNVET  272
10  NYWVSGEIHSLIDNESVSEFKEIIEGGLGILRQES-EEYVARKFEVYATFNNIIPILTT-KNVNEVD-QKFNVLIVNIES  272
11  DYWVSGEVHSMLEFNNVHEFKNIVEGGLKILSQTD-AVSITKRFEIYATFNNIMPVISG-KGSSEIAERKINVLIDHMDI  264
12  DFWVGGEVHSMLDPYNVHEFFNIIDGGLRILSEKT-DECVVKRFEIYATFNNIMPPSST-KGLNDINERKVNVLIDQMEF  264
```

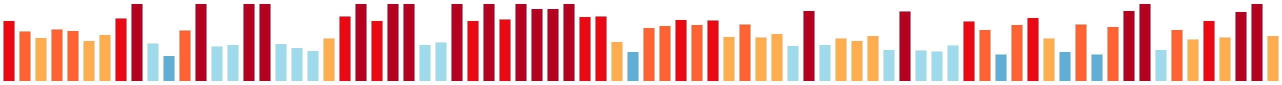

```
IXXXCXPHLXXEQXXLLXXXEKKNPFXXRLYVQIVRFFXXILSNFKIXKWLXKXPXLXXXLKXIYXXSXEALXNENSNKX

1   IIEICIPHLQIAQDTLLSSSEKKNPFVIRLYVQIVRFFSAIMSNFKIVKWLTKRPDLVNKLKVIYRWTTGALRNENSNKI  350
2   IVNVCYPHLKLEQSSLLENVSVKNPFSTRLYIQIVRFFTHLLSNFKVICALSSKRNIQTSMKQVFESSYEALSSPHCNKQ  341
3   IANISIEHLRDEQNSLLQDKEKKNPFSIRLYIQIVRFFTNLFGSFIINKFLISSKALCERMKQVYELSLEALKNENSNKM  376
4   LIKVATLNLQEEQTELLKSHNKKNPFITRLYVQIVRFFGSILSNFMIIKRISTDPELQKELRIIYKISVEALSNEYVNKM  347
5   LIKILISNLKDEQKELLEGNEKKNPFTSRQYVQIIRLLGIMLSNFKIICWLDKRKPLQVMLKEIYKLSEEALVNENSSKV  364
6   ITDVCLPHLDWEQKKLLGTTTKKDPFSSRIYIQIVRFFTILLSNQILVRRLSKRKTLIAKLSTFYEYSIDTLSNSNSNKV  348
7   IIEVCLPHLRTEQEKLLGAGNKKNPFGSRLYIQIVRLFGSLLAHYRVMRALTKLPSLKEQLGEVLELSKEALVHPNANKV  346
8   IVEICMPHLQTAQEKLLSSNEKKNPFIIRLYVQIVRFFATIMSSFKIVKWLMKRPDLVNKLRVIYSWTTDALKNENSNKI  352
9   IIETCMPHLQMTQDTLLSFSEKKNPFVIRLYVQMVRFFGAIMSNFKIMKWLMKRPDLVNQLKVIYKWTNKALKNENSNKI  352
10  IIEVCIPHLLTAQDTLLSSSEKKNPFVIRLYVQIVRFFGAIMSNFKIVRWLTKRPDLVNKLKAIYRWTTDALKNENSNKI  352
11  VVDTCLSHLEEEQEKLLSTNSKKDPFVSRLYVQIVRFLNSILSNFKIIKWLTNKPQLQLKLKRIYRYSIEALTHNNTNKV  344
12  IVNVCLPHLKEEQQKLLSTGEKKDPFSLRLYVQIVRFFNFILSNFKLLKWLSNKPQLQTNIKSIYEFSIEALTHDNTNKV  344
```

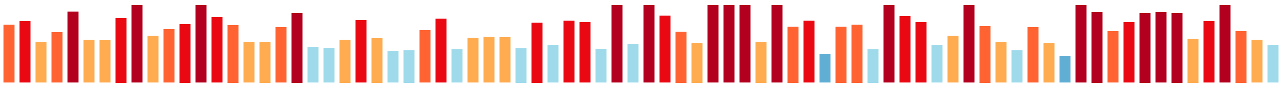

```
   II×AQ××FLRDEKFGT×FL×××EI××II×××T×I×EI×S×NLI×EKL×LIR×FL×KYP×LMI××V××WLPGEVLPRI×××

1  IITAQVSFLRDEKFGTFFLSNEEIKPIISTFTEIMEINSHNLIYEKLLLIRGFLSKYPKLMIETVTSWLPGEVLPRIIIG  430
2  MITAQMIFLRQEKFGTSYLTENERNKLVNIITEVNEIDSYNLKYEKLLLIKALFGKYLKQMLQDVPKWLCGEVLARILVE  421
3  MLICQMTFLRDEKLSYISFTEDEGKHMIQIINQIRDIPSTNAICEKLMLIKQFLIKFPGPMISTIPEWLPTQVISKIVFS  456
4  MIISQLTFLRDEKFSRRYLPMSEITKMINVITQVKDMASTNLICEKLYLVKCFLDKFPVLMIDEIKVWLPGEVLPRILLM  427
5  IIISQLTFLKDEKFSTYYLNKNEILRIIDTFTKIKDLKSTNLMNEKLLLMRCYINKFPKLMIPKISDWLPGELLSKIVLD  444
6  MIISHISFLKEEKFGSYYLPSHTIDNLTSIVPNIKEVDSVNLSTEKLMLLRRFLEKYPDMMFNKASLWLPGEVLLRILLD  428
7  IIGAQYTLLAYEKYGAYFLQKEDVMSIIRSVIRTKDIQSTNLICEKLHLMRKFLSKYTSVMLEIIPEWLPSEVLAQILSD  426
8  IIIAQVSFLRDEKFGTFFLSDEEIKPIISTFTEIIEINSHNLIYEKLLLIRGFLSKYPKLMIETVTSWLPGEVLPRIIIG  432
9  IITAQVSFLRDEKFGTFFLSDEEIKPIIDTFTEIIEINSNNLICEKLLLIRGFLSKYPKLMIETVTSWLPGEVLPRIIIG  432
10 IITAQVSFLRDEKFGTFFLSNEEIKPIISTFTKIMEINSHNLIYEKLLLIRGFLSKYPKLMIETVTSWLPGEVLPRIIIG  432
11 IVAAQVAFLGDVKFGTFFLNDEELTSIIHTIPTIKEIQSTNLICEKLILIKNLLAKYPRLMIENVSIWLCGEVLPRILID  424
12 MVAAQVALLGDSKFGSFFLNDDEITSLIRIIPSIKEIQSTNLINEKLILIRSLIAKYPRLMIDQVSIWFCGEVLPRILIE  424
```

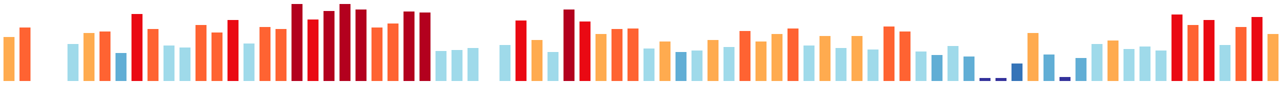

```
   ×E--×YS×KI××T×I×VLLDLLKKCLD×××-×HE×IYQC××××××××××P×K×L×KL××××--×D×-××××××TLG×LLR

1  DE--IYSMKILITSIVVLLELLKKCLDFVD-EHERIYQCIMLSPVCETIPEKFLSKLPLNS--YDS-ANLDKVTIGHLLT  504
2  TQ--TYANKLAVVALEILLDLLKQLLGGKDSVTASIYESMEEKPAKDVLSPSMKMMLQSVL--PDT-YDFETITIGSMLR  496
3  ED--VYSTKTGFTAISVLLELLKRCIDHSI-GHEYVFQCLHIDSTALYFGDSKDKKLQWSNNTLEQTYDINETHLAAILR  533
4  EEENNHSAKIIVTAISIILDLLKKCLDVEK-GHKDIYQCVEIDKVKDVIPRKFLSRIELSL---KEIENVGEKTLGTLIR  503
5  ED--MYAIKITHTSIAVLLDLLKKCLDVEE-GHQEIFKYVEVKTLNESVPEGTLEKFNSTAVLNDLEIDINSMTLGQLLR  521
6  DG--SNGWKCIIAAISVLLDFLRLCLDHDN-TQINIYQSIEVDKLNDVVPQKFLPKMQQYL--KDK-SLLESMTLGQVIR  502
7  EE--VHSLSILQAGIAVVLDLLKKSLDSSA-IHQTINRCIRNTLAKDVMPEGAQNRSVHG-----HSLEWGSTTLEEMLQ  498
8  DE--VYSMKILITSIVVLLELLKKCLDFVE-EHERIYQCIMVSPITETIPEKFLSKLPVSS--HDT-ASLGKVTIGHLLT  506
9  DE--VYSMKILITSIVVLLELLKKCLDFVE-EHERIYQCVMVSPVTETINKKFLSRLPLNS--NDA-GDLNKITIGHLLT  506
10 DE--VYSMKILITSIVVLLELLKKCLDFVD-EHERIYQCVVLSPITETIPEKFLSKLPLNS--SET-ADLSKVTIGHLLT  506
11 SE--INCSKIVLTAVSTILDLLKKCLDVSK-GHEDIYNSVQICTVREVVPAKLLPKLLPSQ--NDP-ESIISQTLGQLLR  498
12 NE--LNSTKIVATAVSTTLDLLKKCLDFSK-GHEEIYRCVEVLFVKDVVPKKLLTKLTPSQ--DDNLDSIAQQTLGQLLR  499
```

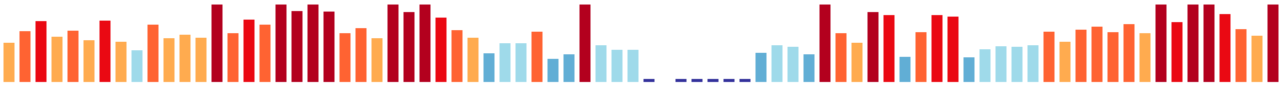

```
   ×QIKY×I××K××YK×AMDLWL×M×GLLYN×××××××L×××-------××××W××LN×LCF×××××K××L×S×KVWRI×TY

1  QQIKNYIVVKNDNKIAMDLWLSMTGLLYDSGKRVYDLTSE-------SNKVWFDLNNLCFINNHPKTRLMSIKVWRIITY  577
2  DHLRNQITEQKAYKIGIDLWLALVGILFNSPRNITKLLPT-----DDRGDSWLSLNELAFKTNDEKAMLLALKSWRVVIY  571
3  DHIDYLVNSKREYKLAMDMWLSFMGLLFNSKGRLAFLASK-------EGEKWVDINLNCLSVNDPKCRILSLKVWRILIY  606
4  NQIKYLILVKKEYKLAMDLWIGMVGLLFNNANSLEMLTKENAETDANRSDTWIDLNKLCYNKGTGKVKYFSSKVWRILFY  583
5  NKIKYLVLVQKDYKVGIDLWLAMVGLLFNNKKNIIHLLDKD-KTHDKSSADWLNLVQLCFKNSDGKTKLLSLKVWRILTY  600
6  DHINYFLLANKHYKIAADLWLAMVGLLYRKADKIEELSAGT------YENNWLSLNSSCFEIPEEHIKLMSLKVWRILFY  576
7  KEITFLIISKKEIKFAMDLWLAMMGLLYNTTEALLELSDT-------DERGWLRLNHICFLSGNSSAKFIALKAWRIITY  571
8  QQVKYYIVHKHDNKIAMDLWLSITGLLYDNEKRVYDLTLE-------SNEVWFGLNNLCFINDHPKTRLMSIKVWRIITY  579
9  EQIKYYIVKKNDNKLAMDLWLSMTGLLYDNGKTVYDLISE-------SSKTWFDLNQLCFMNEQPKTRLMSIKVWRIITY  579
10 KQIKNYIVVKNDNKLAMDLWLSMTGLLYDNGKRVYDLTSD-------SNKGWFSLNNLCFINNQPKTRLMSIKVWRIITY  579
11 KQLKYLILVKQEYKLAMDLWLAMTGLLYNSTDRLQQLTSA-------TDREWLDLNFLCFQADDPQAKLLAIKVWRILTY  571
12 KQIRYLIVAKQEYKLAMDLWLALTGLLYNNIDRLDYLSTH-------IDNEWLSLNLLCFHASSAQAKLLSIKVWRVLTY  572
```

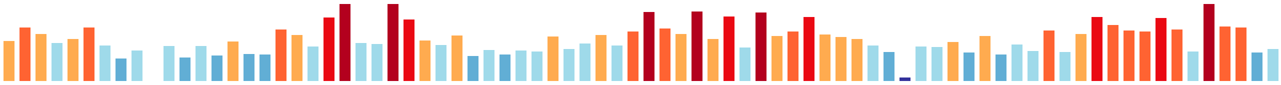

```
    C I C × × I × × × - × × × × × × × L L × L L × × P F × × × × × × × × D × × × R E G × I Y × L × G × V Y T A F × × - × × N × S × × × F × L F W D × L × × P I Y × ×

1   CICTKISQK-NQEGNKSLLSLLRTPFQMTLPYVNDPSAREGIIYHLLGVVYTAFTS-NKNLSTDMFELFWDHLITPIYED   655
2   LVSVNITSLESDEVQLQLLQLLLTPFDQLPNVLEKDCVREGLLYCLNGVVFCSVSMINSLGTQNAISLFWDNLLQPLFHK   651
3   CVTNNIDFL-GEKEAKKISQVLLNPVNVTVNKLEDNSTFMGVKYLLNDFFFSTCGI-TNKCKPTTNIVLWGNFIFPMLAT   684
4   CVSKHLDEF-NETSKPMVLDLLKTPFDMSRPYINDSDVSKALLYCLRGLVYANLCGP-AQSSKESLSLFISNLLKPILIG   661
5   CICTHVTEL-DSAYQIPLLNILKIPLEISKTEHGNDNVRDGIIYYVTGIIYTAFLLLQKNLSYETFQFFFDNLVKPIYEN   679
6   GVCSEISGK-FDEKDLLMLNLLRRPFELSEGQHTMPAVRDGLLYCLNGLTFTIVSTVNVNNDAKRFGFFWDNLLKPLFIS   655
7   YSWAHVQGK-SLHHDHALIRLLKMPFEFSSTSHLEPDVCEGLIYYLTGMVFVTCGN-SKAQEQKTFRFFWENLISPIYHD   649
8   CICTKISLK-NQEGNKRLFSLLQTPFQMTLSHVNDPSAREGIIYHLLGVVYTAFTS-NRNLSTDIFEHFWDHLIAPIYEN   657
9   CICTKISLK-NEERNESLLSLLQTPFQMTLSHVNDPSAREGIIYHFLGIVYTAFTS-NKNLSMDMFNLFWDYLIAPIYEK   657
10  CICTKISLK-NQEGNKMLLSLLQTPFQMTLPYVNDPSTREGIIYHLLGVVYTAFTS-NKNLSADMFELFWDHLIAPIYED   657
11  SICSHLETF-S-TENLRIIKLLQRPFTFSREDQYDFNIMEGLLFHLTGIIYTAFSA-TNNLSTAKFDLFWEHLVRPIYVE   648
12  SICTHLEHE-T-VKNIRIVNLLQRPFEFTQQEQYEFNVMEGLIFHLTGIIYTAFSG-HSTISGEKFSLLWEHVVKPVYMR   649
```

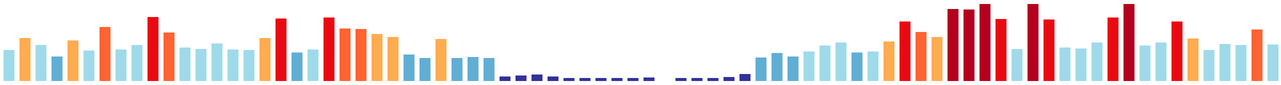

```
    × × F × × × S I × L × × × × × × × L × × L × G G K × × × × × × - - - - - - - - - - - - - - - - × × × × × K × × H P × × V I A S × G V × × × D I × × L P × × × I ×

1   YVFKYDSIHLQNVLFTVLHLLIGGKNADVAL----------------ERKYKKHIHPMSVIASEGVKLKDISSLPPQIIK   719
2   WNSAASSNELQTRTMDIFIKILRGKFPKTSTQRSNS---------PNTKQQQHNFRPVRVIAARGIVISDIPNLSNNFIH   722
3   LLNYSANEDVKFELDTYIGKLLTKAIQN----------------QKNVDKTKSLHPIKVIASEGVSVDDISALQLSQLP   747
4   IILVNQH--LHHEVVSVLLRLLGKPLPETRSPEK------G-------NRNGLRPAPIKVIASTGIATNDISKMTNHLIK   726
5   FIFNSPSFHVQTRLLGILLQLLGAPLFNLVSTSKHITSNAGKSKNIRNDKKVSPNNPVKVIASVGISRDHIVPLPTHLSH   759
6   FLKTSPNNQLRARVIHLLALMMGGHGFDVVKRKRRPGDQEP-YTTKKQKVIRAYADPVRVVAATGISLDDITSLHPNVLN   734
7   FLFTSDSIKLKSAAIKLLAVLIDGKSETGTS------------------KTKSSFPLKVIASVGVTMKEIEPVPADTVD   710
8   YVFLYDSIQLQNILFTILHLLIGGKNNDFAT----------------ERRNKKHIHPMSVIASEGVKVKDISSIPSQIIK   721
9   FVFNYNSIQLQNVPLTVLHLLIGGKNSDVVV----------------ERKNKKHIHSMSVIASEGVKIKDISSLPPSIIK   721
10  YVFKYDSIHLQNVLLTVLHLLIGGKNADVAL----------------ERKNKKHIHSMSVIASEGVKLKDISSLPSQIIK   721
11  RILISQSIQLKARANALLLRLLGGKITEPQH----------------KQVCKKIVHSIKIIASAGVTARDIQPLPLNVIK   712
12  KIFPSTSIQLRNRAIVLLLRLLGSKVMEQQANQQHQQEKQQ-P-HTHRNITKNGAHPIRVIASAGVSARDIQPLSANVIG   727
```

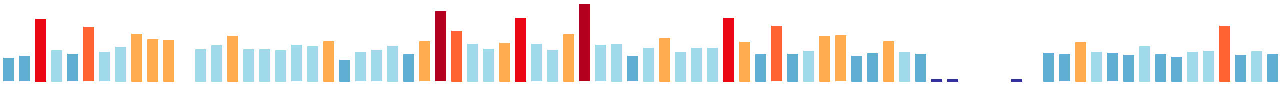

```
    × × Y × × I × × × × F - × × × × × × × S N × × × × × × L I L × × L × × L P × × × × × × × × L E × F × × × × × × × × × - - - - - - - × × × × × × × × × × F × × ×

1   REYDKIMKVVF-QAVEVAISNVNLAHDLILTSLKHLPEDRKDQTHLESFSSLILKVTQ-------NNKDTPIFRDFFGAV   791
2   IWYMQMAKSAS-DLLRTRSFEDEHAKSLFLSLIDTVPESSLNDEVFDYFLLVLKHYLEP------TYDYTFNQLSVLTLF   795
3   YLLETLGSVILLYVLNSKSQNHSQTRMLILEYLEQIPNNQITLSTLCRCISMFYEYYN-------SIDFIANSPRSLISD   820
4   QLYYPLSDLIF-SILRKHILKPQPSSELLISLLNNTPLELKTVSYLEEVNQVMIDILKT-------SDDHTEISLLFQTY   798
5   PGYTLISRLFL-NSLPINAKFLTVKTEIILDLIAQIPDELKTEEVFQEYRNIVVSLTKLYKTSYTTKNKNDVLFECFSRC   838
6   ENYLSIMGLCM-DNTGAHPLS-YLTDKLLYLLLSKLPEHLRNTEVLEAFSDVLSFVVNRS---V-RISSKANTVRIFSTH   808
7   DAYELVLTLVS-NALSIDSSEWATRFELFDSMLRLLPAEFIDDTHLTIFLDLVSRLFS-------QASLPKDPSTVLCPL   782
8   YAYDKIMKVVF-KAVEVAMSNVNLAHDLILTSLKQLPEDKKDQNHLEAFRTLILKVAA-------NNKDKPIFRDFFVTV   793
9   SAYDKIMKVVF-QGVKTAMSNVNLAHDLILTSLKHLPEDRKDQSHLEAFSSLILEVAE-------NNKDKPIFREFFGAV   793
10  SAYDKIMKVVF-QAVEAAISNVNLAHDLILTSLKHLPEDKKDQIHLEAFSSLILKVAQ-------NNKDKPIFRDFYGAV   793
11  SSYEAIKDLVF-QAIESDSSNLCQNFDLITALLKVLPNKFIDKEHLREFTNAIFGVAI-------QRKEDQNLADVFIQI   784
12  TNFETIKDLIF-EAIRSNSSNLSQNSELITALLKQMPSKFVNENSLEDFAEFLIREISI-------QRKDDKNVGSHFVQL   799
```

```
   XXXXXXXFXXLXLXXXXXXXXXXXXXFXXXXXXXXXXXLXLLKXXIXX-XRXXXSEFXIIEXFLX-XXDKXXXXYXXNWV

1  TSSFVYTFLDLFLRKNDSSLVNFNIQISKVGISQGNMTLDLLKDVIRK-ARNETSEFLIIEKFLE-LDDKKTEVYAQNWV  869
2  ATQLVTKFEKLLLEPSNSGLSSVIALYDSKTFINYDFPLKLMISMLDS-NDCDASLFAIFSKFMD-MKNKNYPDYVTNWL  873
3  MSRVCIKFKDIIFDDNYNVFRNFILSFLMLFQEEEHHVDNLLQELIQNALQANISELKLIEGILS-LKKTDCDLFAINWV  899
4  THVVVTIFPNLLLNKETNYLEDYLKLFEPFWGDVKEMLLDVLKDFLRE-KKLAGWEFYMMGIFLK-TKDTLSHLYVENWI  876
5  TCLLGKTFPNIILNPKRDIFSKYLASFDNLFTDNADMLIKLLKDIIKY-TRPQISELTVIELFLTNTNDKNSRDYIQNWV  917
6  SSKLAITFKDILFDKNSKAFTNYIAKFNMLSDREGTCLLTFLKQLLNN-SKGIIPELFVVENFLL-IGNKEVDSYLSNWV  886
7  VLSLTGPFANFVFESSK-NFQKLLKGVDHFYGQDSEIRLTLMKEFSKK-LRRQISEFTIIERFLE-VGDLSCQRYASNWI  859
8  SSSFVYAFLELFLKKHDSSLVEFSIQLSKVGIPQGNMTLDLLKDVIKK-ARNETSEFVIIEKFLE-LNDKKTEVYAQNWV  871
9  GSSFIHTFLDLFLRKNDSSLINFNIRFSQIGSPQENTTLNLLKDVIRK-SRNETSEFVIMEKFLE-LKDKKTEIYAQNWV  871
10 IPSFVYAFLDLFLRKHDSSLINFNIQFSKVGILQGNMTLDLLKEVIRK-ARNETSEFLIIEKFLE-LEDKKTEVYAQNWV  871
11 TCCLATQFATLFSDERK-IFEEYLGKFSFILLANGETKIKLLKELIVG-LRGKMSEFFIIEAFLQ-QGDLSTKEYVSNWI  861
12 TCCLMSQFTPLLFEDRK-NFEAYLRKFEFIFSVNPDTKVKLLKELIMG-LRGKVPEFFLIESFLE-PQNGAIRKYVSNWI  876

   GSTLLXPXXXXXEFXSLANIVXXXPXXXXIENFLDLCXKXXXXVNXXXLLXXXXWXXXXXXXFIKXYXSKNXNXLXXXLX

1  GSTLLPPNISFREFQSLANIVNKVPNENSIENFLDLCLKLSFPVNLFTLLHVSMWSNNNFIYFIQSYVSKNENKLNVDLI  949
2  SVAVLPSNMNRHDMKFLMNIISKNPLVSIIENLLTVIADIDREFYGMICTSALTWDDHALLYFIKGVFTSNAPNSCNYL-  952
3  GKKLLSPSVQEGDFILYSRIVAKISEKQTIVNFLDLADKLGFEVNPTEILRISFWKPSGLVIFLEEYIAKHHVDFADIVS  979
4  GSTLLSPTMSYTSFLDLANIINQITTDQVMENFLDLCSKMSLSINYPQLLNLKQYNDKNLSFFIKTYLSKNSNALDNNMS  956
5  GSTLLSPMLTYPEFQSLTNIVNMIPSSQIVENYLDLCVKTSLAQNPCTTLNIRNWKDKQLIFFIKTYISKCSNRLDDDIL  997
6  GSTLFPPSLSKDEIIALAHCVSTIHTEKAIENFIDYSKKIE-QLDVVTLLHLGAWDESDFCFFVKAYLAKRKNVSVTHLI  965
7  GSVLLPNTLTTSELHSFASIVKLLLSPQAIGALLNFCLNSKDKPDICELLNIDDWGDEKVAILVKAVVSLSSNKLVPSLA  939
8  GSTLLPPNISFKEFQSLANIVDKVPNENSIENFLDLCLKLSFPVNFFTLLHVSKWSNNNFIYFIQRFFSKNDEKLNADLI  951
9  GSTLLAPNISFKEFQSLAGIVDQVPNEHSIENFLDLCLKLTFPVNLFALIHVSKWSNDNFIYFIQSYVSKNNNKLDTDLV  951
10 GSTLLPPNISFREFQSLANIVDKVPNENSIENFLDLCLKLSFPVNLFTLLHVSKWSNNNFIYFIQSYVSKNENRLNADLI  951
11 GSMLLSPTMPHEHFSTLVNIVTLIPTTEVLENFLNLSSKVNYDVDLFSLLNIQNWEFQGIIIFIKNSILKNHNTIKPELN  941
12 GSMLLSPTIPYEHFCSLVRIVNAVPTTEVLQNFLNLTSKVPFEVDLFHLLNLKSWDDKELTLFLKNSIIKNLKPNRPELY  956

   XXLXXXLPXNX----XLFXXLLPXLXXXKXXXIXXXXIXXNPXXLNXXXXXXXX----XLXXXLPXXXXSYFXXNXXXF-

1  TLLKTSLPGNP----ELFSGLLPFLRRNKFMDILEYCIHSNPNLLNSIPDLNSD----LLLKLLPRSRASYFAANIKLF-  1020
2  ---SGCLPTRISKSLFLFKAILNSPELFKENGIVLRSVLKNPSLIQNLNVAEVT----KLRDLSDHDAPTDFMKYIN---  1022
3  SIIDADRLNNV----ELFSYVLKHLPENQKSAFIKQHYTKCPLIWNQGVLIDDY----DLIDITLDCEICTFIAHHFSFL  1051
4  QFLIEILSENRTS--SVPRILLPLLIDIKRYDIIKTSIEQRPLILSIVSDPILL----HLKDILPTSKMSYLIINIGLF-  1029
5  VLLHDVLPDNI----DVFDSMVPLLIQINRTDLIRHCIEKKPSYLNTPAIMNYH----DLSEILPDDRKCYLLNNLKDF-  1068
6  ALLQKLLPSRP----ETFTELLPTLLEYGHKELVSDCVKKNPILIESLLPKYPD----NLSDFFSADEILAFVPDMKNF-  1036
7  VKLQKMLPSRV----SLFNELFTTLQTLKYEELIRHVIIANPEAANGIILLNGTQAPAVLPKGLPRDTLSYFVSQLPNF-  1014
8  TLLKTSLPGNP----ELFFGLVPFLRLNKFIDILKYCICSNPALLNSITDLNSD----FLMQLLPLSRASYFAANIQLF-  1022
9  TLLKTSLPGNT----ELFVGLLPFLKEKKFMSILEYCIQSNPTLLSYISDLNSD----LLLELLPRSRTSYFVTNIQSF-  1022
10 TLLKTSLPGNP----ELFFGLLPFLRNNNFIDILEYCICSNPTLLNSIPDLDSD----FLIKLLPRSRASYFAANMKLF-  1022
11 LFLQSVLPNST----PIFSEMLPFLCGAKHYNIVRYTLTKSPKFLNLTTSVDKK----SFDEILPLEILPFFLQSISDY-  1012
12 SFLENILRENE----QLFSTLLPLLCQLGHYEVLRLVLTHRPTFLSHTLGVDEE----SFDQILPLEKLSFFLKNLLNY-  1027
```

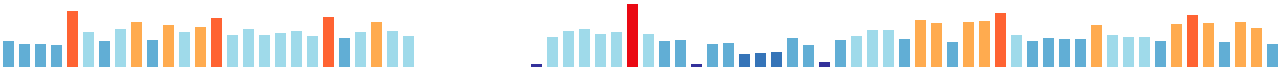

```
   XXXXQLXXIXWXLXXXXXXXLXXNXX--------XXXXXLXXX-XXXXXXX-XXXXXXIXXLLXXXXXXXXXXLLSXLXX

1  KCSEQLTLVRWLLKGQQLEQLNQNFS--------EIENVLQNA-SD---SE-LEKSEIIRELLHLAMANPIEPLFSGLLN  1087
2  ----------VVYKKQIAGTMNNKLSQSGTSTDKSNSECFNEPQLDNQGQTGEDTSVVVLEKLHECCSNNKGDALTEILS  1092
3  STSSKAKLLIIFIEKDMYDLLVQFRY-------NLIQEFLPTLTIYKKD---DIITTVQQHLINYCFAKNSFVLLDCIEP  1121
4  PQLIQYGIIQWVLEKDETALLLSNYA--------TIETILFVD-KPTDTSVNEERKKLIQHILHYLFENRRWVLLSNLID  1100
5  SAIVQYKIIMWVAKTDEFTLFKKNYV--------KIESCLFLD-EPCSENILD-KKAFIKELFTIFYEKQAWQLVAILVV  1138
6  SSETQVILLKEAWEKSGLSTVVKNFD--------SVASCLFEQ-ALDG-SISNVRKILTDSILSSIIKDERWDLLNNFFV  1106
7  DENAQLGMIKWILDMNEADLLFADRG--------EWKDCVFLR-IGKDGEFSNEFKILIADAISQLYEKQSWKNLSKCIL  1085
8  NNSEQLTIIRWLLKGQLLEEINQNFS--------EFKSSLQNA-SG---HE-LERSEIIRELLLLTAKNPIEPLGGLLE  1089
9  NHAEQLTIICWLLEGEELDQINQNFP--------EVKNILLHT-PA---DE-LEKSKIIGELFRLATKNPIEPLFSELLN  1089
10 KSSEQLTLIRWLLKGQQLDQLNQNFS--------EIESVLQNA-PD---SE-LEKSEIIGELLCLTTRNPIEPLFSGLLY  1089
11 QDTIQLRILQWCIEHNQINMLFGHYE--------TLEHYLFKK-EYGTEES-SRRNQLLLDLLSSLLVGKKWLYMSKIIQ  1082
12 ETFFQLRTIRWALKHNQINILFENSS--------VLQKCLFQQ-CTSSEDT-MKKEKLLLDLLSVTVLGEKWHYLSLLVI  1097
```

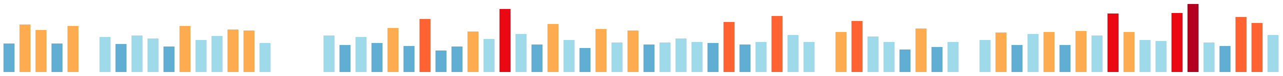

```
   XCXXN-XXXXXXXXFXX---XXXXEXLXXXXPXXLXXXXXXXXXXNXXLXX-XXXXXXXX-XXXDXXXELXXXLIXXKXX

1  FCIKN-NMADHLDEFCG---NMTSEVLFKISPELLLKLLTYKEKPNGKLLA-AVIEKIEN-GDDDYILELLEKIIIQKEI  1161
2  NLLSQ-DQTTIISNFFCSYAAQNIEVIKLISPKLLRHLCKFYPDHLHALILPAINYLFGPVQKISTLTSLAIILIEKKDI  1171
3  STLTS-RTLEDVIKLLE---KSYEDNITFVPAKYIIFIDNLPMDTKINTTE-ILRVYLVT-GDVTQCLFFLTSLINDKKM  1195
4  SCLSN-SQTDYIISLFS---GGNATELQNILPQTLANMINRCGSLNPSLID-TLKQNYSL-QKADYIITLISELVECGKT  1174
5  SCINH-GYESNATEFLA---NIDSNTLNSFPPKAIATIVNRCGSMNPKLLL-ILHQFFSN-KEKFFLLELVSELISLKKI  1212
6  TAYLNGRDTGLLDNFFT---KVDSESLEHFQPNFLVAICDRLGY-SERRKN-VLKKCFFV-KEKEYVISLIEALIKSKKF  1180
7  YCMTC-GQVPCVEQVYV---RMRDKRRENNSPK--SPKADECKSLNSILSE-SIKEIYQG-DAIDTAFEVTTSLIELKKV  1157
8  ICVEN-SIIDCLNEFCE---AITDELLLKINAELMIRLLTYRDNPNEALLT-AVTERIRN-ADSDYTFEFIGKIVARKEI  1163
9  FCIDN-SMTNNLDEFCE---TINDEILSKINPELLMRLLTYRDKPNVTLLT-VLAGNIGN-ADSDYKFELLERIIARKEL  1163
10 FCLKN-SMTDHLDEFCG---KVTDEVLLKINPELLLKLLTYNDKPNGKLLT-AVTEKIGN-ADNDYILELLEKIIAQKEI  1163
11 SCMKN-GQASPVTKTFS---KNDPESLFWLTPVTLASMINKCGLLNPSLIE-VLKKCFSR-KPADFNIELTKELINRNES  1156
12 WCMEN-NEIGHLTTCFS---KENLTPLIHLSPQALALMINKCGSLNPTLIE-AVKRSFFT-NDTQFNIGLIEELIVHNKF  1171
```

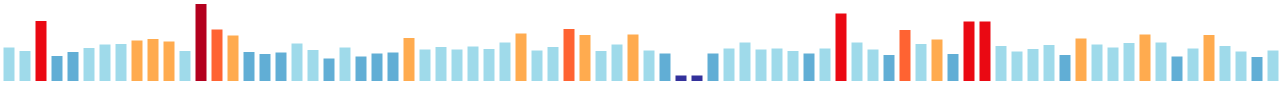

```
   XXLXXXXXXXXXFFXXXXXXXXXXXXXXXXXXXXXLXXXLXX--XXXXXXXXFXXXLXSXLPXXXXXXXXXXXXXLXXXX

1  QILEKLKEPLLVFFLNPVSS-NMQKHKKSTNMLRELVLLYLTKPLSRSAAKKFFSMLISILPPNPNYQTIDMVNLLIDLI  1240
2  NGLDRIIDPFSIFLFKGPKKLSSDIKHKIAGLFLKMFFLLKE--ERGVHLSIVLHRIVRSLPRRRPEYLKNVINTISTQK  1249
3  VPLTRCRNEVFDFFIALDSSNDLSIKAKAVTTFESLINVIFL--LRKKVDIGFLSQYIARLPERPSRYLVDLSEKLILNK  1273
4  QALILSKQDMLLFIIDSENALRPVDQRRILELMPRFIQSLQN--KSEKTFIDFLKMLFNLLPEHSNQYLLNLVQLFKESL  1252
5  DALLSVSSELINFFIDDQHIFTEDEQGKSIGILEKFLAQLQK--YNERTSFNFTKDLMSSLPKSKTKYLSKLASCVGSAQ  1290
6  DVLNDLRSEFITFLLVHSKDPSLTFEKQYVDGINNFILLLKE--QSEDVVVDFMVYLISPMQKQMSINDKTVLKYMISDE  1258
7  QNFSSCREEFLNFFIIRASNFVSVEQAIALELFERFIAVLME--CFDEIILSFVESILSSILKVLTDHSLQVFGLLNHHL  1235
8  LLLEKLKEPLLVFLLSPVRS-SEQKYEESANMLRELVLLYLTKPLTRSAAKKFFSMLVSILPSNPNHQTIDMVYLLIDLI  1242
9  LVLEKLKESFFIFLLDPVSRLNKENHDNGTKIFCELILLYLTKPLSRGAAKKFFSMLVSTLPERPDRQSIDLVHLLIDII  1243
10 QLLEKLKEPLLVFFLNPVRS-NKQKHEKSANMLRELVLLYLTKPLSRSAAKKFFSMLISILPPNPNRQTIDMVALLIDLI  1242
11 QVFFLCGKEVVAFFTVRSKMFTLSEQDAISALFEMLMTSLMC--QKDSLILDFVQELYANIPQECDSHLLKILIIAVKKL  1234
12 QLFILCGKEIVTFFLDNSSSLSGVEQDGVLSIFEKLLVSLMG--QKKSLVLDFVEEISEKLPEAPDAYLLKIITILVEQL  1249
```

```
   XXXXXXXKXXXXXXXXXXXXXXWXXXXXXXX---XXXXXXXXXXXXXX----XXXXXPXXXXX--X--XXXXXXXXXXSX

1  KSHNRKFKDKRTYNATLKTIGKWIQESGVVH---QGDSSKEIEAIPDT----KSMYIPCEGSE--N--KLSNLQRKVDSQ  1309
2  NLE----NKIPDINDIQLALTKWIKLEDYSSYLLKYRRYAYLDQITNQVGITQTNRQPHSN-----------GIVGTNIA  1314
3  SFNRSKFRNSSSFNDLHTHVRTWCNQLDNTSGS-----PRETTPIS----------AAHIN----D--------------  1320
4  PSSFKNSKSRREMGRLNKNFQRWKDETKSIEPTSG------EQAISSE----STMSTPE-GFDKST---KSQNSSSNDNA  1318
5  NST--------VVGVTQEELKLFSQKLEDCD----------NQEVNKT----LAIVQPKAQFVVSRDIRDVYTNKIQDSD  1348
6  YIKNVSIKRIPKFDDLVDHLIALE----------KQDSSNLTMTSFSD----SSLSTPEGLQ-------ISNNKNSEESA  1317
7  TLKGHLFHKLENYKTLLEKSGESKLVKENVEAQCSNGGPTLLEPIH-T----GTTSNASDMGQDKN--QSDDINKAAVSY  1308
8  KSHNRKFKDKRTYNATLKRIGKWIQESEVDQ---QEDSRKEIDTTYSA----NSINIPDKESE--K--NVLNSQRGSNSE  1311
9  KSHNRKFKDKRTYNATLKTIEKWIHDSEVVL---QRDPDKEAGEKLND----SSIYTPQGLEVEKG--VPENLQKTPNIE  1314
10 KSHNRKFKDKRTYNATLKTIGKWIQESDVVH---QGDSSKEIDTTPNA----NSTYVPHGESE--K--EVSNLHRKADFE  1311
11 QAKGKHLEKYDRYHHISGELFSILSNLTITS---QNVGLK------------NGIAQETPQ----N--NESN-FTEKNSK  1292
12 QVNGKKLKKENRFSQALQRLVELHYN-----------GIK------------NQSYEDHHE-------NQER-IRETQSS  1298

   XIQVPATQGXXXXXXX---XXXX-----------XXXXXXXXXXXXXKXXXXXXXXXXXXXX--XXXXXXXXXX---XXX

1  DIQVPATQGMKEPPSS---IQIS-----------SQISAKDSDSISLKNTAIMNSSQQESHA--NRSRSIDDETLEEVDN  1373
2  QIEVPEMQSLSKTKDNAAIEEGSAQQGVET-----------------------------------LI---TG-----TNS  1351
3  HINKNALSLEKDKDNL---TNN---------------------TEQQKNIPNSKS-------------------------  1351
4  EIQVPATQLKQDNDVT---TGTTTAKSAYSYQFKSSTEDESTDVLSQKSTGIIKSPEE-------LIQPTKNTH------  1382
5  EIQVPATQKDEEKEII---AIGSQSQSNNDLNNSSIEEENKNNTIVSKNLAIVTSIETPSGTQSQEIRQLTGNH------  1419
6  EIQVMGTQDVNKISGI---SNMR-----------AIH------------SPQVNFSDSRHST--KIIASSPNVVNGQDRN  1369
7  IIEVPATQGISKSAES---TEGR---------------------------------------------------------  1328
8  DIQVPATQGIKDLPSS---LQLS-----------SQNDIKSSHTIKLKKFPITNLAQPEILA--EISRSSTNEFLGEADG  1375
9  EIQVPATQGVKDLPSS---FQTP-----------SQADVEDFYVRKVKNIPIMKLTQREIPA--KLGRSFTDETLEEVDN  1378
10 DIQVPATQGIKDPPSS---YQLS-----------SQLDAKNSGTIKLKNIPIMNLAQQETLA--KRSRSFADETLEEVDN  1375
11 ISMNPQRRGL----------------------------------------------------------------------  1302
12 SENVPTPKGFNDPRIS---ESP---------------------------------------------------------S  1318

   XXXXX-XXXXXXXXQXXXX-XXXXXXXXXXXXXXXXX----XXLXEXXXXXXX---------XXXXXXXXXXXXXXXXXX

1  ESIRE-IDQQMKSTQLDKN-VANHSNICSTKSDEVDV----TELHES-IDTQS---------SEVNAYQPIEVLTSELKA  1437
2  IGMQE------NFSQEPLNDSI-DRTVHSHEGNGTQATYLANALSRIDNEQNHS--------TKQHNASTSNSLTSTLSE  1416
3  --------------QEISK-FS---------SNIADA----NDLPSNTRESNC-----------DG-SQSTEPAGGKMIK  1391
4  --------------------YQ-------EEKAPLVS----QELPSYRSSQDPRSTQEGKITNGDGTAIPDSSIIGTLEE  1431
5  --------------------LE-------NESK--EV----SDLPEVYS---PSDT--------VNKRIDDSSFTQTLDE  1455
6  LAMKKENKVPTISGTEDKKIFVNSSSDIISCSDDAHH----LNLFEGDKNANR---------LDDSSEEQMKNF------  1430
7  -------D-----SQRDMN-IVTSTIIHD----TVE----------------S---------TEISSFVKMDVASQK---  1363
8  ESTGE-GDQQANTTQLDDK-TINDKSIYSTNNGEMQV----TELQEYS-GVHP---------SKANSDQQTEVVIDKLEG  1439
9  ESMGE-IDQQAKSTQLNNE-IDNHKDIQYNKSDDAEV----SELHEDDATANS---------SE----------------  1427
10 ESARE-IHQQMKSTQLDEN-TVNRTSIHFTKSDETEV----SELHEDDNDTHS---------SGANAYKQTEVFTGELRE  1440
11 -ESHDRLEIQVYATQTKTS-QIEE---------PVAV----EEEGKNNEGRRF---------STDHSVAEKKTTTAE---  1355
12 EKIQEINEVQVYATQEKHK-VQNE---------PVKL----SHQTEGQVSLVA---------SEP--SSDK-----K---  1365
```

```
   XXX--XX------------XXXXXXXXXXX-XXXXXXXXEXXXXXXXXXX-------------XXXXX-XXXXXXX----X

1  VTNRSIK-----------TNPDHNVVNSD-NPLKRPSKETPTSENKRSK-------------GHET---MVDVLVSEEQA  1489
2  VTS-----------------YGGLIQSDTQEVRT----------TLNK---------------ESS-IDDDIVD-----  1447
3  ID------------------KGQISAD------------------------------------MKNNKIA-----  1407
4  VDKQIAK-------EPVTQSTGCGQLENH----TNIVGESSDLPEVLEDEN-------------AGSIIKDDLLSTENRN  1487
5  VEDPENVNMIQAKSTQIGPTTGDTQINNI----SDTNAEAPDLEDKVDEEK-------------QE-------------  1504
6  ----------------------------------VERQEGEKENVKEKFRQGSASKKSERQRDIAHAGSKTN-DRLRPQNDS  1477
7  -------------------------------------A-------SEEVAR-------------EHESS-------------  1375
8  VKKELVK----------TSLDQNLVDND-DLVQCFPGESPVLEDKRPK-------------DHEKDKKMGDVSSFEEEG  1494
9  ----------------------------------------------------------------------------------  1427
10 VTNKFFK-----------PNPDHNSVNSD-NFLQRSAEETPISEDKTSK-------------GHEE---AANVLPPEEEA  1492
11 -----TR-----------ESPQEIIVQSNTNPNK----EAPE-------------------DAASAKTM---------G  1386
12 -----NR-----------PILEEAVI----DPR---------------------------SG--R---------G  1382
```

```
   ----------X---------------XXXXXXXXXX---------------XXXXXXXXXX---XXXXXXXXX---XXXX

1  ----------VSPSSDVICTNIKSIANEESSLALR---------------NSIKVETNCNENSLNVTLDLDQQTITKED  1543
2  ----------L---------------SQKSSPVE--------------------AVGTTCV-----------------  1463
3  ---------------------------------PR---------------QISSIASEPDTL---VNSNIDNKSMIEKGKN  1437
4  DTEIGNELVVAEPTPTVVEAKIATVSEQVIPMTPSKTNPQTDAETPFQTPRSSLGVEPHASEKDAAKTIDSDD----KTN  1563
5  ----G------------SSNLYTVGNVVLPFACSPEQRGVDN--IQQGEISTLSTSRNLSIEDTGSSYSNQE---KGED  1562
6  ----------T--SSDI--------TNTKGNFAVK---------------N-------------------NKEIMKTYR  1502
7  ----------------------------------------------------------------------------------  1375
8  ----------VSSSTELVPTIVKSVSQQLSTI-PK---------------GSEKADLNCSGNLSTITLSSNDQTMTNKN  1547
9  ----------------------------------------------------------------------------------  1427
10 ----------VSLSTEVVRTNVKSVANEESLLTLR---------------NSQKIDPKCSERSLTATLSLDKQTVTEKK  1546
11 ----------S-----------------------------------------------------------------------  1387
12 ----------L-----------------------------------------------------------------------  1383
```

```
   GXXXXXXX---X-XXXXXXIXX--XXXXXXXXXXXXXXKX----------XXXXXXXXXXX-XXXXXXXXXXXXXXX---

1  GKGQVEHVQRQENQESMNKINS--KSFTQDNIAQYKSVKK----------AR-PNNEGENNDYACNVEQASPVRNEVPGD  1610
2  ---------HLP--NNIHEIDI--DKEEN-----TTPKKE-AQMTI--NTQAVNES----N-SATNLEVRLSALPIE---  1514
3  GDKTVNNN------ATTND-------FEY--RTSEDPI----------------------------VSVNIPIFNSR---  1471
4  GF--EERL---PTNQTSQEVVTDLGGERG--SPHAKSVKSNDSIIVQSKRANKNNTNEEPNDIECVEGKSGQA-------  1629
5  GASKSSRS---PHR----EIETHVEDEHV-TVPELNMHK--HSIHIISN--NVTENMEQPRNKTHIEGTTISKEKKGLED  1630
6  GHGDLMTS-----PVRFYEIEK--D--DGIPISVSSPIKR----------QKINFLLPNSN----VIERNL---------  1550
7  -----------------------DGFSLTPVAQAIPVTY----------PVAQTS------------------------  1397
8  GKEQRERTDNLEKQDSINNINK--ESFMQDKITQNEPVKE----------TALQISEDEGKYRTCIVERALPIINAELSD  1615
9  ----------------------------------------------------------------------------------  1427
10 GNEQEEKDQSLEKKESMNIIDN--ESPTEDNITQNKTVKK----------TASHNNENEDKNWTYNVEQTLPAMNEGMAD  1614
11 --------------ASQNILKV--QNFKVS---KVSKVKN----------PDVAFI-----------------------  1414
12 --------------SSEDAIDV--EDLEPESRRENSNMHS----------PDILTN---------NLET-----------  1417
```

```
   XXXXXXXX-XXXNXXXX-XX-XXXXXXX---XXXX---X-------XXX-----XXXXXXXXXXXXXX-----XXXX---

1  GIQIPSGT-ILLNSSKQ-TEKSKVDDLRSDEDEHGTVAQEKH-QVGAINSRNKNNDRMDSTPIQGTEEE----SREV---  1680
2  -----TGTQMIDNSNKY-------KG-------SEMVTGSDLRAEGKVS-------------------------------  1544
3  -----------------------VSGLD------------------K---------------------------------  1477
4  ------T-------------------------------G----------------QSFKANPSKSSAD--SDKPTNLDTK  1654
5  MTENSSI-------------------------------G----------------EAKLALPMATIVDDPQSRSTNVDTE  1663
6  --KATQETEISNNGNATDIVPIEVNGDHMDNDGAVSLAGNAT-GGGKNN-----NVVIEAVTPNSESDR----GRHV---  1615
7  -----VEE-ITTNKHKD--------A-----QDPG---------------------------------------------  1413
8  GLQNSSGI-MLVNGLKQ-KEGSKVDTLNHGEDEKVTFAEEDP-QMISVNSSEENAQSIDSVPMQNAVKG----SVEA---  1685
9  --------------------------------------------------------------------------------  1427
10 GIHTSSEA-IFLNNSKQ-TEKSKADALHYNEDEHGMATGENR-QSDEINSVNQNAKKIDSIQIQSVEEE----SRKA---  1684
11 SSTSCPKT-PFTTGSSK-NV-TEINDAV---LE-------------STRSLERGDD------------------------  1451
12 QISSNGKG-SFLNGNGK-AL-LPYDAVR---DA-------------RNRDINENEEH-----------------------  1455
```

```
   XXXXXXXXXXXXXXXXXXXXXXXXXXXXXXXXXXXXDDFXPXXX------X-XXXXXXLX--XMXXXXSXX---------

1  VMTEEGINVRLEDSGTCELNKNLKGPLKGDKDANINDDFVPVEE------N-VRDEGFLK--SMEHAVSKE---------  1742
2  ---------KTDRSGSII-----------TRNSNVNH--RPGPE---------NSSGLLTLAIYKSTASR----------  1583
3  -----------------------------LNNQKISDEYVISSHDLELARNGLQNQAQLH--DMHEGYKKKSANLLTQ--  1524
4  EMEEDNLKSRNELRTALDEKETKSRTYIEGEEDKGEDDFAPVNT------A--QQDPFLE--AMEKEVSQTEVIL-----  1719
5  ERENSTTSKSSHLIGAL------------EEQQATQDDFVLVEE------P--PKDTLLD--ELDKEISQDMTET-----  1716
6  RISSDSITNKV-------SRNDDFVPVESTRNSSDNDDFVPIEA------A-IEEDNFIE--EMEKEAIRN---------  1670
7  ------------------QKKGANIDKKKEATVESNDDFAPAET------D--QDDEFLL--AMEQKLETTSNKLAPIIG  1465
8  ITTEETLTVHLRDSETTEFNKMMERQQRKGQKINTNDDFAPVDE------N-DRNEGFLE--AMEQVASRE---------  1747
9  --------------------------------------------------------------------------------  1427
10 VTIEESLSERLEGCGTLEPIKNLEGQLKEDKKANINDDFVPVEE------D-GRDEGFLK--AMEQVASKE---------  1746
11 -------F--------APVNVAP------------KDDFLE------------EMEEKIK--IKNQKSSEQ---------  1481
12 ------VFSRI--LGATEFRTFP------------KDEARETKE------VREEKTDNLE--ERPQTVKDD---------  1498
```

```
   -------XXXXXXXEXXXXSXXXXIRIPIFNSXKXXXXXXXXXXXXXXXXX----XX--------------XXXXXX---

1  ------TG-LEEQPEVADISVLPEIRIPIFNSLKMQGSKSQIKEKLKKRLQ--------------------RNELMP---  1792
2  --------------EAPNTQFAPEVHF---GPKKVEPVTSVKMIK---RDQGSMGDIV---------Q----E-------  1623
3  -------HNMRPPFEDVAIPTHKELKIPIFNSLILKSTNQQPMIPNSQRKV--------------------QSEVAI---  1574
4  -------KSSPIAKNKRPTSSMHEINIPIFNTLKLTDPSWKERSRTQQESTTRMGQQKQIDSSQVTST---RS-----D-  1783
5  -------NASTAHKDVVLDSPMPAIRIPIFNTLKIGSGRIEKKKHTKIDKA--LNKRLNLDSSAIILH---QQEDAPCDV  1784
6  -------------LERPAKHHIPSIRIPLFNVSRPSGRENTKQQKDNTHTG----AISTFSDVGVKINEGNFNTIED---  1730
7  QNPPEQNKLQSVSLDDVDAPASSKIRFPIFNSSKFQGRSVKVLNNTTNLTQ----NA-------------K---------  1519
8  ------TSLLKEQTELADTSVLPEIRIPIFNSLKIQESNGQIKQKLKMRLQ--------------------RNESMP---  1798
9  --------------------------------------------------------------------------------  1427
10 ------TSLLKEQPEVADISVLPEIRIPIFNSLKIQESKSQIKEKLKKRLQ--------------------RNELMP---  1797
11 --------IEGIEANHNDVSQMSQIRIPIFNSSKLQNKLDLEGPRCDKGE------------------------------  1523
12 --------DAMASVENKDESQVLGIRIPIFNSSKLQNKPKSKDSVHENDLS----ER-------------KRKRVDG---  1550
```

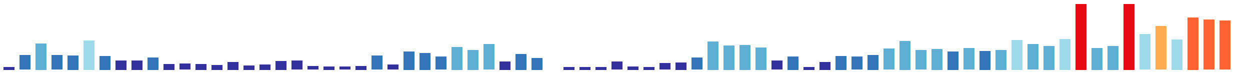

```
     ----XDXXPXXXX----X-XXX----XXXXXXXXXXX-------XXXEXXLXX-XXXXXXXXXXXXXXXXDXXSXEXTPS

1    ----PDSPPRMTENTNINAQNGLDTVPKTIGGKE-KH---------HEIQLGQAHTEADGEPLLGGDGNEDATSREATPS   1858
2    ---EKDVNTAFEP---FQQDAAIAKV------------ALKDDEMSCEIQ-----------VSENENCADDTSSKEGTPS   1674
3    -----------------R-KR-----PLGNPSTQGQY--------------------TDIENERVRDYDDSQSLEDSAD   1610
4    ---KQDVIP------------------------------------SHLEVDLHS--SFPYGTPGNGIDDDMDINSRETTPS   1823
5    PIITKDHLPEEIDTVT-KQAPAFGNIPVNNMAEE----------VPLENDLQS--SFVIDG-----KEDGDGTSRDTTPS   1846
6    ----KKMNPSFNESTEVL--AG----KTNSKGMEARKDEFKDDIEDDEIE-----SSSSEDRMQDDEEYRDEASSASTQS   1795
7    ----------------------------NADEKEPDN-----------------NARSNGQTLEESRCSEDAVSRDATPS   1554
8    ----PDSPPRVASNANTNNVLN----DSTSVGKEGKY---------DEIQLAQSHIEKDGDPLLVGEGNEDATSREATPS   1861
9    --------------------------------------------------------------------------------   1427
10   ----PDSPPRLAANTNIIGRNGLGTAPKAIGGKEEKH---------GEIQLAQAHTEADADPSLVGDGNEDATSREATPS   1864
11   ------ESPREPR-------------D-SSVGKACDK----DAKNSQAIDLES-SQEDNESNILDNNSKEDY-SKEPTPI   1577
12   ----NDEEGNDYD-------------E-HEVETAREV----DPISTQPEDLAS-S-PDNESTFLQDSRRSDWASQNSAPS   1606
```

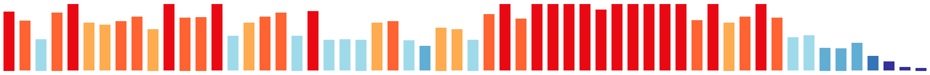

```
     LRXHFPSKKXRKLVXRLRXFXXXDLXXXSXEEXRNLRIELLDFMMKLEYXXXXXXX----

1    LKVHFFSKKSRRLVARLRGFTPGDLNGISVEERRNLRIELLDFMMRLEYYSNRDNDMN--   1916
2    LHIHFPSKKARKIVNRLRDFKPDQLAALSAAERRNLRVELLDFMMKMEYFEAEE------   1728
3    IRLHFPNKRARRIVSKLRGFSEYDMGQLSAEEKRNLRIELLDFLMKIEYHTITD------   1664
4    LRMFFPSRKTRRLVSRLRNFDPSELSTLPTEERRNLRIELLDFLMKLEYYTGDDADKREI   1883
5    LRMHFPCRKSRKLVNRLRTFNPDELASLPVEERRNLRIELLDFMMKLEYYTGDKNN----   1902
6    LRDHFPSQNARKLVRYINELGHEELLQFLPEEKRNLRIELLDFLMKLEHYSDK-------   1848
7    LRMHFPSKKARKLVNRVRAFTAEDLSHLPPEEKRNLRIELLDFLMSLEHEHGFYQ-----   1609
8    LKIHFSSKKSRKLVSRLRGFTPGDLNGISVEERRNLRIELLDFMMRLEYYSNRDDDMV--   1919
9    ------------------------------------------------------------   1427
10   LKAHFSSKKSRRLVGRLRGFTPGDLNGISVEERRNLRIELLDFMMRLEYYSNRDNDMG--   1922
11   LRLHFPSKKARKLVSRLRGFTVEDVSDISPEEKRNLRIELLDFMMKLEHESLNGEF----   1633
12   LRLHFPTRKSRKLVSKLRGFSLDDISRISLEEKRNLRIELLDFMMKLEHESLAEEL----   1662
```

Consensus Threshold: >50%

Compare to: Saccharomyces_cerevisiae

Amino acids that match the reference are marked with orange highlighting.

Created: Aug 10, 2020

Last Modified: Aug 10, 2020
